# Estimated prevalence of post-intensive care cognitive impairment at short-term and long-term follow-ups: a proportional meta-analysis of observational studies

**DOI:** 10.1186/s13613-025-01429-z

**Published:** 2025-01-10

**Authors:** Mu-Hsing Ho, Yi-Wei Lee, Lizhen Wang

**Affiliations:** 1 School of Nursing, Li Ka Shing Faculty of Medicine, The University of Hong Kong, 5/F, 3 Sassoon Road, Academic Building, Pokfulam, Hong Kong; 2 Sijhih Cathay General Hospital, New Taipei City, Taiwan

**Keywords:** Cognitive impairment, Critically ill survivors, Post-intensive care syndrome, Proportional meta-analysis, Systematic review

## Abstract

**Objective:**

Evidence of the overall estimated prevalence of post-intensive care cognitive impairment among critically ill survivors discharged from intensive care units at short-term and long-term follow-ups is lacking. This study aimed to estimate the prevalence of the post-intensive care cognitive impairment at time to < 1 month, 1 to 3 month(s), 4 to 6 months, 7–12 months, and > 12 months discharged from intensive care units.

**Methods:**

Electronic databases including PubMed, Cochrane Library, EMBASE, CINAHL Plus, Web of Science, and PsycINFO via ProQuest were searched from inception through July 2024. Studies that reported on cognitive impairment among patients discharged from intensive care units with valid measures were included. Data extraction and risk of bias assessment were performed independently for all included studies according to the Preferred Reporting Items for Systematic Reviews and Meta-analyses reporting guidelines. Newcastle–Ottawa Scale was used to measure risk of bias. Data on cognitive impairment prevalence were pooled using a random-effects model. The primary outcome was pooled estimated proportions of prevalence of the post-intensive care cognitive impairment.

**Results:**

In total, 58 studies involving 347,940 patients were included. The pooled post-intensive care cognitive impairment prevalence rates at the follow-up timepoints < 1 month, 1–3 month(s), 4–6 months, 7–12 months, > 12 months were 49.8% [95% Prediction Interval (PI), 39.9%–59.7%, n = 19], 45.1% (95% PI, 34.8%–55.5%, n = 23), 47.9% (95% PI, 35.9%–60.0%, n = 16), 28.3% (95% PI, 19.9%–37.6%, n = 19), and 30.4% (95% PI, 18.4%–43.9%, n = 7), respectively. Subgroup analysis showed that significant differences of the prevalence rates between continents and study designs were observed.

**Conclusions:**

The prevalence rates of post-intensive care cognitive impairment differed at different follow-up timepoints. The rates were highest within the first three months of follow-up, with a pooled prevalence of 49.8% at less than one month, 45.1% at one to three months, and 47.9% at three to six months. No significant differences in prevalence rates between studies that only included coronavirus disease 2019 survivors. These fundings highlight the need for further research to develop targeted interventions to prevent or manage cognitive impairment at short-term and long-term follow-ups.

**Supplementary Information:**

The online version contains supplementary material available at 10.1186/s13613-025-01429-z.

## Background

The combination of higher intensive care unit (ICU) survival rates and an aging global population is likely to result in a greater need for ICU resources. With the proportion of elderly individuals in the population expected to rise to 20% by 2050, the demand for ICU care is likely to increase, highlighting the need for effective critical illness and post-intensive care syndrome (PICS) management [1, 2]. PICS affects cognitive, physical, and psychological sequelae that impact the quality of life and persist beyond hospital discharge among critically ill survivors. It can include a range of symptoms, such as muscle weakness, cognitive impairment, mental and emotional distress, as well as post-traumatic stress disorder [3]. PICS is a significant issue for ICU survivors, and there is a need for clinicians to screen for it and for policy development to address its impact [4]. The Society of Critical Care Medicine (SCCM) defined PICS as “a new or worsening impairment of cognition, mental health, or physical function after critical illness that persists beyond the acute care hospitalization [5].” PICS is considered to have a multifactorial etiology and is associated with inflammatory conditions, for example, sepsis and acute respiratory distress syndrome (ARDS). During the COVID-19 pandemic, there was a significant increase in the number of critically ill patients developing ARDS. COVID-19 infection has been shown to cause COVID-19 ARDS, which can result in patients receiving mechanical ventilation and ICU-level support. This association between COVID-19 infection and ARDS highlights the potential for COVID-19 to contribute to the development of PICS in affected patients [6].

One of the primary features of PICS is cognitive dysfunction, which can be attributed to various factors. Prolonged stays in ICUs, the need for mechanical ventilation, use of sedative and steroid medications, use of physical restraints, prolonged immobility, and underlying comorbidities are all potential contributors to PICS development. These factors can interact and exacerbate each other, leading to long-term cognitive dysfunction that may persist even after hospital discharge, underscoring the importance of addressing PICS risk factors in ICU patients [7]. Older age, have a mental illness, or neurological diseases are at a higher risk of developing this condition [8, 9]. The mechanisms of cognitive impairment in critically ill survivors are not yet fully understood due to the diversity of ICU patient populations and disease diagnoses [7]. Cognitive impairment in critically ill patients is a multifaceted issue that likely involves a combination of various risk factors, including comorbidities, genetic factors, and exposure to specific ICU-related factors [10]. Hypoxemia, the duration of hypoxemia, delirium, hypotension, glucose dysregulation, pre-existing mental health problems, inflammation, and cytokine activation are all potential contributors to ICU-related cognitive impairment [11, 12]. However, these factors and their interactions have not been fully elucidated. It is unclear whether cognitive sequelae in ICU patients can be ameliorated [10, 11]. Currently, there is no summary evidence of the overall estimated prevalence of cognitive impairment among critically ill survivors discharged from ICU at short-term and long-term follow-ups. A review of 19 studies reported a wide range of cognitive impairment in 4–62% of patients during post-ICU periods ranging from 2 to 156 months [10]. The prevalence rates varied greatly, and the review did not explicitly highlight a specific time to follow-up, as it included studies that evaluated patients more than two months after ICU discharge. The prevalence of short-term follow-up (less than two months) remains unknown. Given that there is an increasing number of the prospective studies investigated the post-intensive care cognitive impairment in a longitudinal follow-up time period. This meta-analysis aimed to estimate the overall prevalence of post-intensive care cognitive impairment among critically ill survivors discharged from the ICU, considering regional differences across studies, and individual characteristics.

## Methods

The guidelines including Meta-Analysis of Observational Studies in Epidemiology (MOOSE) [13] and Preferred Reporting Items for Systematic reviews and Meta-Analyses (PRISMA) [14] were followed in this systematic review and proportional meta-analysis (eTable 1). The review registration number on PROSPERO is CRD42024570155.

### Search strategy

A comprehensive search was conducted across multiple databases such as PubMed, Cochrane Library, CINAHL Plus, EMBASE, PsycINFO via ProQuest and Web of Science. The search was performed using MeSH terms and relevant keywords such as cognitive dysfunction and intensive care units, and included all articles from the inception of the databases up to August 2024. The complete search strategy is outlined in eTable 2. Additionally, a manual citation chasing was performed to identify any relevant articles that were eligible.

### Eligible criteria

Inclusion criteria for the studies were as follows: (1) observational studies with retrospective and prospective cohort, or cross-sectional design reporting on the prevalence of cognitive impairment; (2) patient population consisting of ICU survivors who were admitted to and discharged from ICU; and (3) identification of cognitive impairment using validated measures and available to define cognitive impairment or diagnosis by physicians. In the case of multiple articles utilizing data from the same patient source, the study with the most comprehensive information or the largest sample size was included to avoid overcounting the prevalence of post-intensive care cognitive impairment. Exclusion criteria were as follows: (1) editorials, reviews, letters to the editor, discussion paper, or conference abstracts with insufficient prevalence data; (2) failure to provide an operational definition of cognitive impairment; (3) observation of cognitive impairment prevalence in ICU without post-ICU follow-up observations; or (4) publication not in English language.

### Study selection and data extraction

The title and abstract of all records identified in the literature search were independently screened by five reviewers, including MHH, YWL, and three research assistants with experience in conducting systematic reviews. Remaining articles’ full texts were independently reviewed by the same five reviewers. Each article was assessed by at least three reviewers independently. To ensure consistency and accuracy, we created a data extraction sheet in a standardized format for reviewers to record study details such as publication information (first author, year of publication, and sample size), country/region, study design, where the study was conducted, COVID-19 patients involved, illness severity scoring systems including acute physiology and chronic health evaluation (APACHE), simplified acute physiology score (SAPS), sequential organ failure assessment (SOFA), and cognitive function measures, as well as sample characteristics including mean age, percentage in male, and the number of cases developed post-intensive care cognitive impairment. The information was extracted independently and reviewed for consistency and accuracy. The research team reach a consensus through discussion if there were any discrepancies.

### Risk of bias assessment

Two reviewers (MHH and YWL) assessed the risk of bias of included studies using the Newcastle–Ottawa Scale [15]. This scale comprises three sections, which assess sample selection, comparability of study groups, and exposure/outcome assessment. The scale includes questions about the representativeness of the sample, adjustment for confounders, appropriateness of exposure/outcome measures, and adequacy of follow-up or participant response rates to determine the risk of bias in a study. Assessment criteria were detailed in eTable3. The final decision of studies inclusion and exclusion was made through discussion with the research team.

### Data synthesis and analysis

The Freeman–Tukey double arcsine transformation of proportions was adopted to pool prevalence estimates in this proportional meta-analysis [16, 17]. The follow-up timepoints for the cognitive function assessments were varied, and several studies have reported multiple follow-ups across time, thus the follow-up timepoints were categorized into five timepoints [< 1 month, 1–3 month(s), 4–6 months, 7–12 months, > 12 months] for understanding the prevalence of post-intensive care cognitive impairment in a short-term and long-term period after ICU discharge. Given the diversity of patients undergoing various treatment procedures and diagnoses, as well as the inherent heterogeneity of prevalence data, we anticipated substantial between-study heterogeneity. Therefore, this proportional meta-analysis was performed using a random-effects model [18]. In situations where multiple assessment tools were used to measure cognitive impairment, prevalence data were obtained for meta-analysis from the most frequently used assessment tools. The pooled prevalence estimates are expressed as percentages, along with 95% prediction intervals (PIs).

To assess statistical heterogeneity, we used Cochran’s *Q* test and *I*^*2*^ statistics, where *I*^*2*^ values of 75% suggested high heterogeneity [19]. The predefined subgroups, including the geographical location of the study (continents: Asia, Europe, North America, Oceania, and South America), study design (prospective, retrospective, or cross-sectional), sample size (< 100 or ≥ 100), and studies focus on COVID-19 survivors only were used to explore potential study-level factors contributing to heterogeneity in the prevalence of post-intensive care cognitive impairment. To reduce the family-wise type I error resulting from multiple comparisons within subgroups, we applied Bonferroni correction to the significance level.

To evaluate the robustness of the pooled prevalence estimates, we conducted sensitivity analyses by excluding studies. We also performed leave-one-out analyses to determine the impact of outliers. Egger’s test was used to evaluate small-study effects. A significant level of 0.05 was set. All statistical analyses were conducted using Stata 18 software.

## Results

### Search strategy and study characteristics

**Fig. 1:**
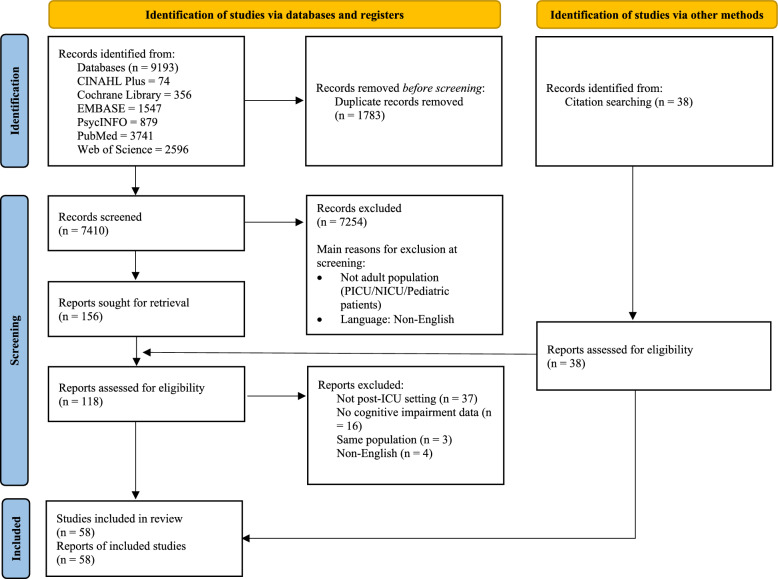
PRISMA 2020 flow diagram

The initial search produced a total of 9193 records from all databases, which 7410 records were retained following the elimination of duplicates. From these, 58 studies that were deemed eligible according to the pre-specified inclusion criteria, and had been published between 2006 and 2024, were ultimately selected for inclusion in this review (Fig. 1). Of all studies, 75.9% (n = 44) used a prospective cohort design [20–63], 17.2% (n = 10) used a retrospective cohort design [64–73], and 6.9% used a cross-sectional design (n = 4) [74–77] for reporting the quantitative results. Most studies were conducted in North America (39.8%, n = 23), followed by Europe (36.2%, n = 21), Asia (17.2%, n = 10), Oceania (3.4%, n = 2), and South America (3.4%, n = 2). The most common measurement for cognitive function was the Montreal Cognitive Assessment (MoCA) and a total of 22 studies (37.9%) adopted MoCA to identify post-intensive care cognitive impairment. A total of 347,940 patients were included, with the mean age ranging from 42.7 to 83.5 years, and 38.5–84.3% of these patients were men. Table 1 shows the key characteristics of the included studies.

**Table 1:** Summary of key characteristics of included studies (n = 58)

Author, year	Location	Study design	Sample size	Male (%)	Mean age	Illness severity scoring systems	Cognitive function measurements
Balasubramanian 2020	India	Prospective	136	66	56 (median)	APACHE: 26 (median) SOFA: 12 (median)	RBANS Update questionnaire
Bladwin 2021	US	Prospective	185	48	74	APACHE II: 29	CAM-ICU and Mini-Cog test
Bark 2023	Sweden	Prospective	57	77	61	SAPS III: 53	MoCA
Bottom-Tanzer 2023	US	Prospective	126	64	53	APACHE II: 21 APACHE III: 67	Clinical examination by physicians
Brück 2019	Sweden	Prospective	100	76	54 (median)	APACHE II: 26 (median) SAPS III: 48 (median)	IQCODE (screening), CANTAB, CFQ
Brück 2018	Sweden	Prospective	216	61	62 (median)	APACHE II: 8 for no sepsis; 13 for severe sepsis (median)	CFQ
Bulic 2020	Australia	Prospective	103	52	60	APACHE II: 20	MMSE
Carenzo 2024	Italy	Prospective	105	78	61	APACHE II: 10 (median) SOFA: 4 (median)	TMT-B and MoCA-Blind
Castro-Avila 2023	Chile	Prospective	252	65	57 (median)	NR	MoCA–Blind
Chung 2017	South Korea	Retrospective	30	70	61 (median)	SOFA: 7 (median)	Mini-Cog test
Costas-Carrera 2024	Spain	Prospective	102	70	66	APACHE II: 13 SOFA: 6 (median)	MoCA, Digits forward and backward from the WAIS-III, Stroop Test, FCSRT, JLO, TMT, COWAT, BNT, CRQ
De Tanti 2023	Italy	Prospective	29	65	60	NR	ACE-R
Dubin 2021	US	Prospective	165	48	65 (median)	NR	MoCA
Duggan 2017	US	Prospective	280	53	59 (median)	Modified SOFA: 4.8 (median)	BRIEF-A, subjective measure, and TMT Part B
Elias 2024	US	Retrospective	49	67	73	APACHE III: 81.5	The NIH Toolbox Cognition Battery, FICAT, DCCST
Fagerberg 2023	Denmark	Prospective	64	52	65	NR	WAIS III, WMI, PSI, POI
Fernández-Gonzalo 2020	Spain	Prospective	156	62	63	APACHE II: 17 (median) SOFA: 7 (median)	NART Spanish version, Subtest of Digits, Spatial Span, Symbol Search from WAIS-III, RAVLT, BVRT, SCWT, TMT, FAS test
Ferrante 2018	US	Prospective	391	41	84	NR	MMSE
Fjone 2024	Norway	Prospective	684	70	60 (median)	SAPS II: 31 (median)	The Mini MoCA
Geense 2021	Netherlands	Prospective	3320	66	63	APACHE IV: 62 for medical; 58 for urgent surgical; 50 for elective surgical	14-item CFQ
Godoy-González 2023	Spain	Prospective	80	69	60 (median)	APACHE II: 8 (median)	IQCODE (screening), Digit Span Forward and Backward from the WAIS-III, Spatial Score Forward and Backward from the WMS-III, RAVLT, SPART, SCWT, TMT Part A and B, CTT, FAS, NART, Vocabulary subtest of the WAIS-IV, PDQ, CRQ
Guerra 2012	US	Retrospective	25,368	48	77	NR	ICD 9th edition
Habib 2014	Pakistan	Prospective	138	84	54	NR	MMSE, McNair's and Kahn Auto-evaluation (Urdu translation)
Haddad 2020	US	Prospective	1040	60	62 (median)	APACHE II: 24 (median)	CDR (screening), IQCODE
Hatakeyama 2022	Japan	Prospective	334	80	68 (median)	SOFA: 5 (median)	IQCODE
Jackson 2011	US	Prospective	173	57	43	NR	IQCODE
Jaquet 2022	France	Prospective	41	76	56 (median)	SAPS II: 30 (median)	MoCA
Jones 2006	UK	Prospective	30	57	54 (median)	APACHE II: 16 (median)	CANTAB
Kang 2024	South Korea	Prospective	475	59	61	APACHE II: 12 SAPS III: 33	MoCA-Blind
Karnatovskaia 2019	US	Prospective	300	57	53	APACHE III: 32–49 (median) SOFA: 4–8 (median)	MoCA-Blind
Kawakami 2021	Japan	Prospective	192	65	74 (median)	APACHE II: 23 (median) SOFA: 8 (median)	SMQ
Kim 2023	South Korea	Cross-sectional	131	59	64	APACHE II: 18	The Korean-MoCA
Klinkhammer 2023	Netherlands	Prospective	205	70	63	APACHE IV: 55 SOFA: 7 (median)	MoCA, TMT, Stroop, COWA, Category Fluency, Digit Span, Symbol Digit Substitution, RAVLT, JLO, BNT
Ko 2022	South Korea	Retrospective	306,011	65	58	NR	ICD
Kosilek 2021	Germany	Prospective	289	67	64 (median)	NR	TICS-M
Maley 2016	US	Cross-sectional	45	42	59	NR	HUI-3
Mart 2024	US	Prospective	501	59	50 + (median)^a^	SOFA: 5 for lower SpO_2_; 4 for intermediate and higher SpO_2_ (median)	Telephone MoCA
Martillo 2021	US	Retrospective	49	73	54	SOFA: 4.5 (median)	Telephone MoCA
Martínez 2023	Argentina	Prospective	40	75	69 (median)	APACHE II: 19 (median) SOFA: 8 (median)	MoCA
Mason 2024	US	Retrospective	134	76	65	NR	MoCA
Mateo 2022	Spain	Cross-sectional	29	55	63	SOFA: 3 (median)	MoCA
Mimenza 2024	Mexico	Prospective	107	42	70	NR	MoCA
Mitchell 2018	Australia	Prospective	148	69	57 (median)	APACHE II: 18 APACHE III: 57	RBANS, TMT Part A and B, MMSE
Nordness 2021	US	Prospective	590	59	61 (median)	SOFA: 6 (median)	RBANS, TMT Part B
Palakshappa 2021	US	Retrospective	696	49	75	NR	ICD 10th edition
Pereira 2018	Portugal	Prospective	267	56	62	SAPS II: 44 SOFA: 3 (median)	DRS-2
Proffitt 2023	US	Prospective	50	52	59	NR	MoCA
Sevin 2018	US	Prospective	162	48	54	SOFA: 10 (median)	MoCA, TMT Part A and B
Sturgill 2023	US	Retrospective	94	51	53	SOFA: 11	MoCA
Sylvestre 2019	France	Cross-sectional	40	58	41 + (median)^b^	SAPS II: 43 for non-ECMO; 42 for ECMO SOFA: 8 (median)	WAIS-IV
van Sleeuwen 2024	Netherlands	Prospective	2476	62	61	APACHE: 56	abbreviated CFQ-14
Vialatte de Pémille 2022	France	Prospective	13	39	62 (median)	NR	MMSE, FAB; 40 words oral naming test, Dubois five words test, forwards or backwards digit spans; similarities test of the WAIS-IV, Brixton test, SCWT-Victoria version; categorical and lexical verbal fluencies during two minutes
Vincent 2022	Switzerland	Prospective	156	83	63 (median)	APACHE II: 25 (median) SAPS II: 58 (median)	CPC, mRS
Weidman 2022	US	Retrospective	87	74	62 (median)	SOFA: 12 (median)	MoCA or MoCA blind (for video visits)
Wilcox 2021	Canada	Prospective	150	54	57 (median)	APACHE III: 57 for surviving patients; 75 for patients who died or withdrew (median)	RBANS, TMT Part A and B, IQCODE-SF
Wood 2018	Canada	Prospective	70	69	67 (median)	APACHE: 19 (median)	RBANS
Yanagi 2021	Japan	Retrospective	248	70	69 (median)	APACHE II: 16 (median)	Mini-Cog test
Yao 2021	China	Prospective	431	59	55	APACHE II > 15: n = 158 (39%) SOFA > 4: n = 31 (8%)	MoCA

*APACHE* Acute Physiology and Chronic Health Evaluation, *SAPS* Simplified Acute Physiology Score, *SOFA* Sequential Organ Failure Assessment, *RBANS* Repeatable Battery for the Assessment of Neuropsychological Status, *CAM-ICU* Confusion Assessment Method for the intensive care unit, *MoCA* Montreal Cognitive Assessment, *IQCODE* The Informant Questionnaire on Cognitive Decline in the Elderly, *CANTAB* Cambridge Neuropsychological Test Automated Battery, *CFQ* Cognitive Failures Questionnaire, *MMSE* Mini-Mental State Examination, *TMT* Trail Making Test, *WAIS* Wechsler Adult Intelligence Scale, *FCSRT* Free Cued Selective Reminding Test, *JLO* Judgement of Line Orientation, *COWAT* Controlled Oral Word Association Test, *BNT* Boston naming test, *CRQ* Cognitive Reserve Questionnaire, *ACE-R* Addenbrooke’s Cognitive Examination Revised, *BRIEF-A* Behavior Rating Inventory of Executive Function-Adult, *NIH* National Institutes of Health, *FICAT* The Flanker Inhibitory Control and Attention Test, *DCCST* Dimensional Change Card Sort Test, *WMI* Working Memory Index, *PSI* Processing Speed Index, *POI* Perceptual Organization Index, *NART* National Adult Reading Test, *RAVLT* Rey Auditory Verbal Learning Test, *BVRT* Benton Visual Retention Test, *SCWT* Stroop Color and Word Test, *FAS* verbal fluency test, *WMS* Wechsler Memory Scale, *SPART* Spatial Recall Test, *CTT* Color Trails Test, *NART* The National Adult Reading Test, *PDQ* Perceived Deficits Questionnaire, *CRQ* Cognitive Reserve Questionnaire, *ICD* International Classification of Diseases, *CDR* Clinical Dementia Rating, *SMQ* Short-Memory Questionnaire, *TICS-M* Telephone Interview of Cognitive Status, *HUI-3* Health Utilities Index-3, *DRS* Dementia Rating Scale, *FAB* Frontal Assessment Battery, *CPC* Cerebral Performance Category, *mRS* modified Rankin Scale, *NR* not reported, *ECMO* Extracorporeal membrane oxygenation

^a^Median age in the lower SpO_2_ group: 55 (n = 142), intermediate SpO2 group: 58 (n = 186), and higher SpO_2_ group: 51 (n = 173)

^b^Median age in the non-ECMO group: 51 (n = 18), and ECMO group: 41 (n = 22)

### Risk of bias assessment

The risk of bias assessment result is summarized in eTable 3. The methodological quality of all studies deemed satisfactory, and no study was excluded due to the risk of bias assessment. Most studies were rated as having good quality, the variations in scores were primarily attributed to the absence or ambiguity of adjustments made for confounding factors.

### Prevalence of post-intensive care cognitive impairment

**Fig. 2:**
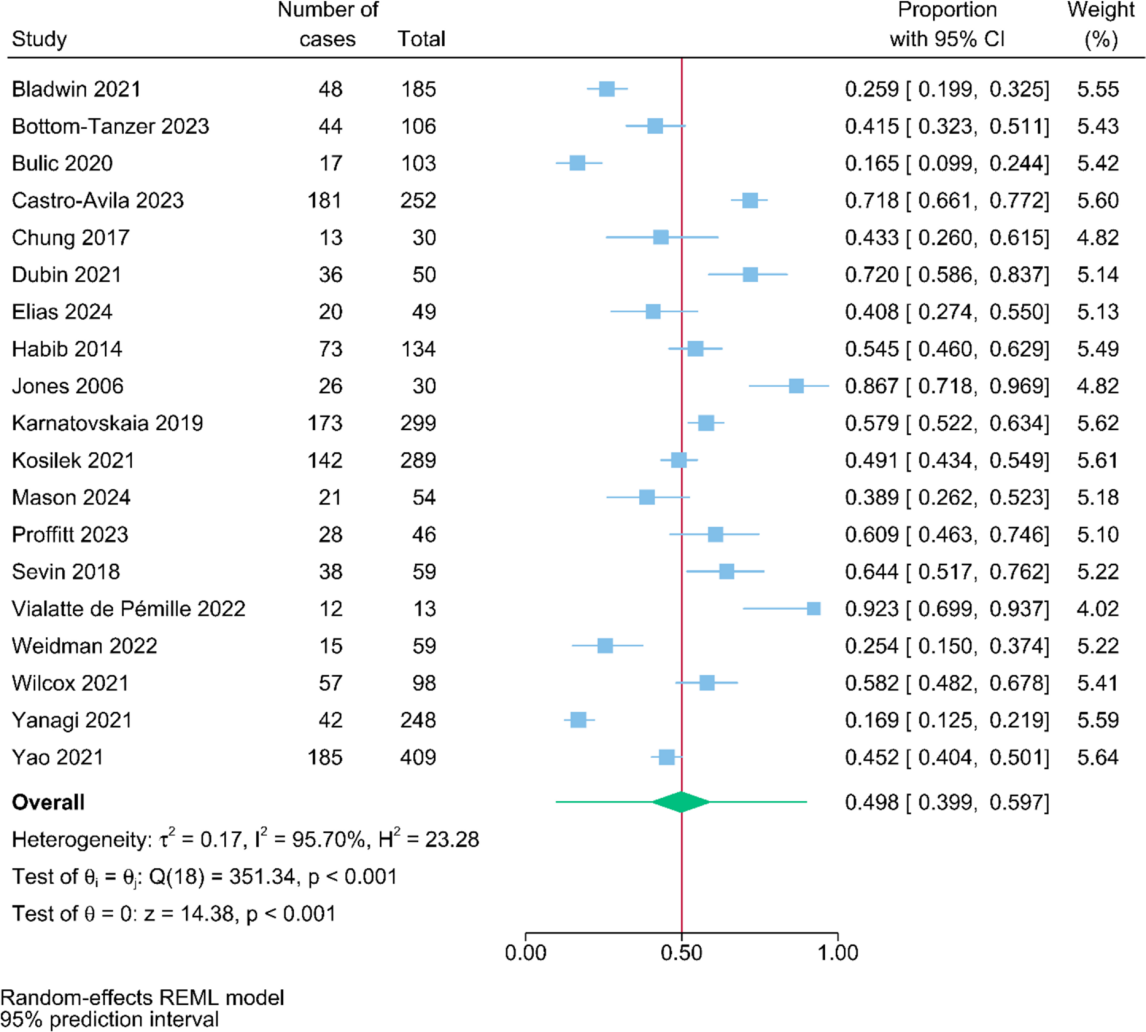
Pooled proportions of post-intensive care cognitive impairment at follow-up within a month

**Fig. 3:**
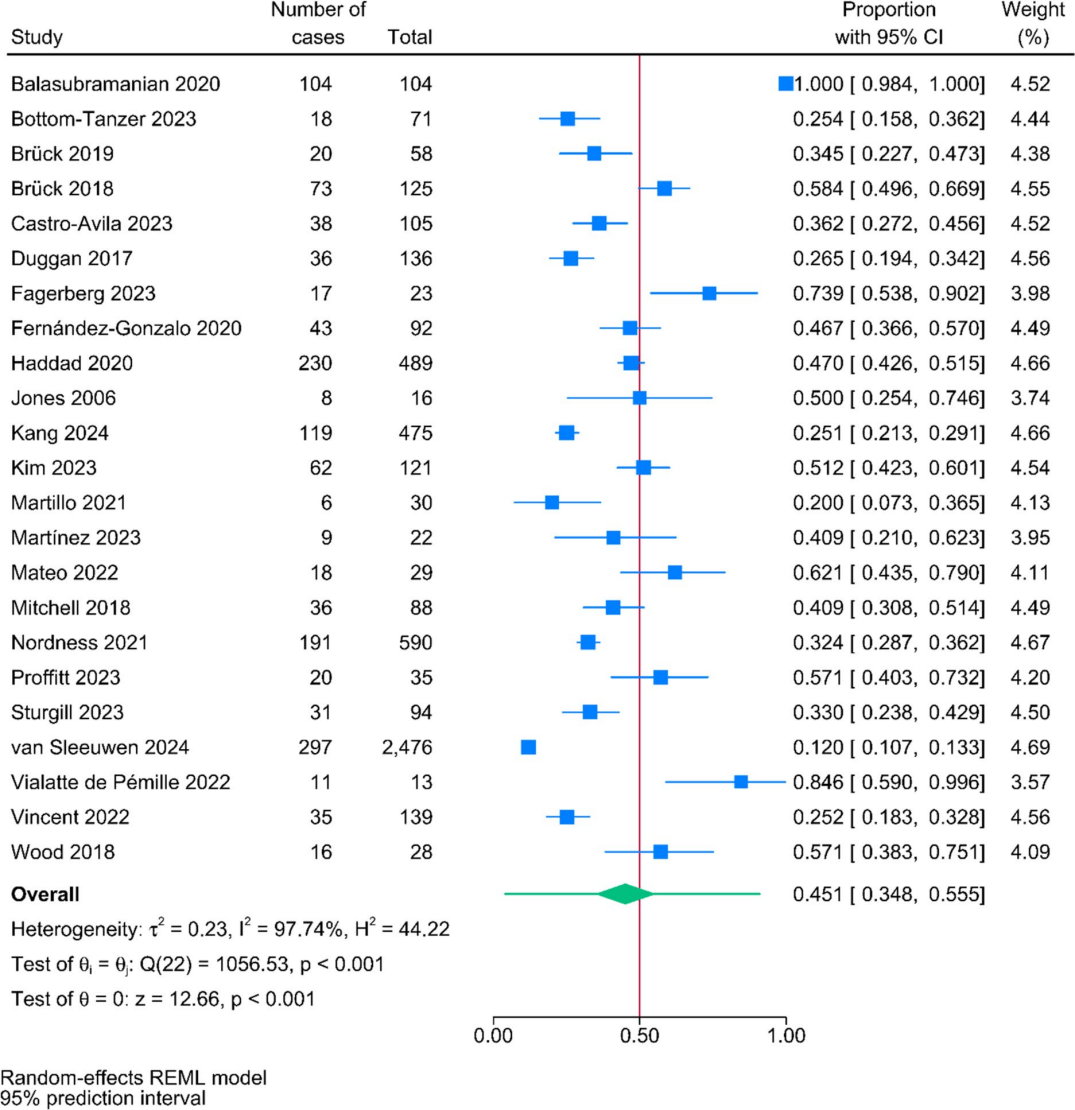
Pooled proportions of post-intensive care cognitive impairment at 1 to 3 month(s) follow-up

**Fig. 4:**
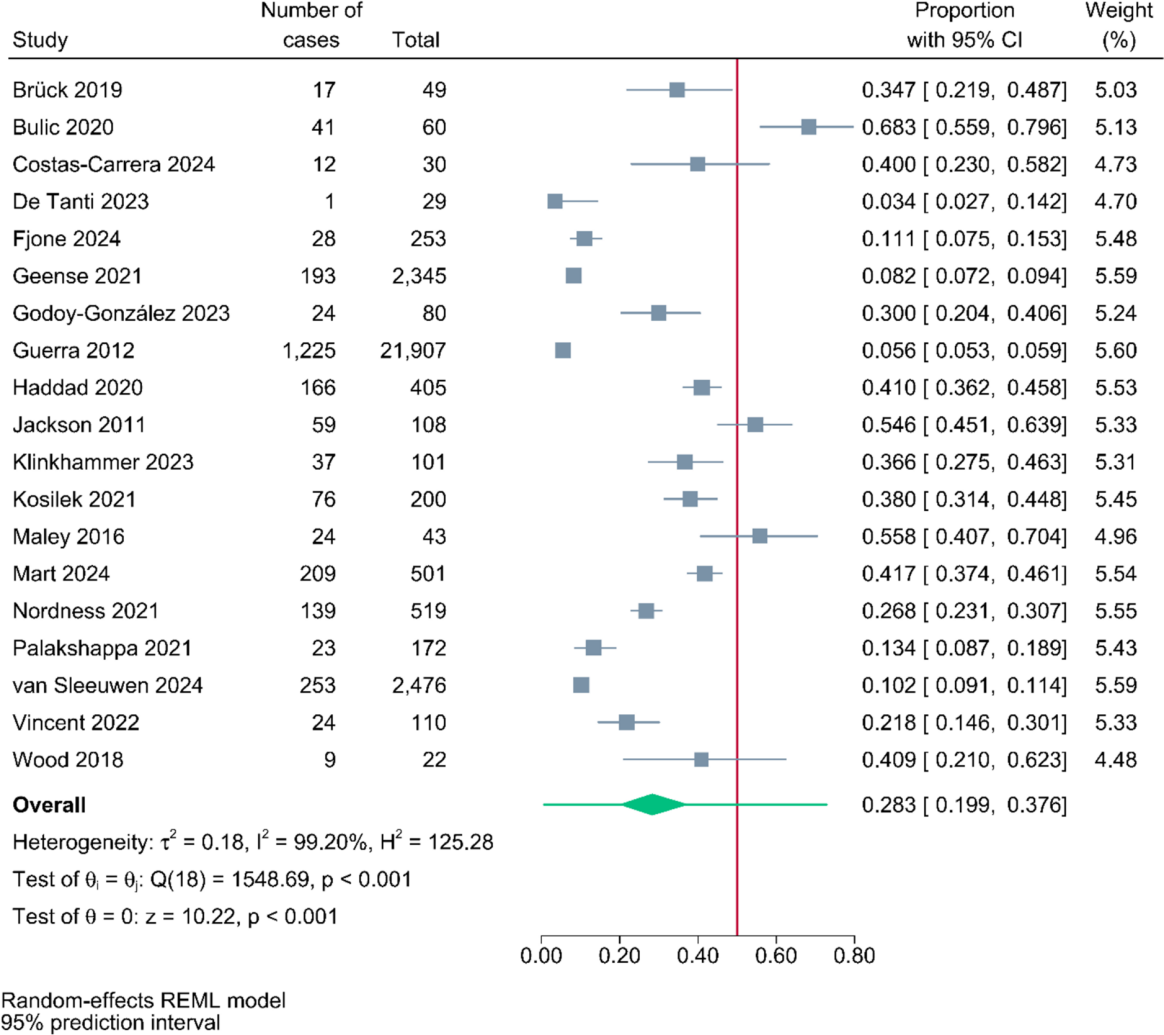
Pooled proportions of post-intensive care cognitive impairment at 7 to 12 months follow-up

The pooled prevalence rates of post-intensive care cognitive impairment at the follow-up timepoints < 1 month, 1–3 month(s), 4–6 months, 7–12 months, > 12 months were 49.8% (95% PI: 39.9%–59.7%, n = 19; Fig. 2), 45.1% (95% PI: 34.8%–55.5%, n = 23; Fig. 3), 47.9% (95% PI: 35.9%–60.0%, n = 16; eFigure 1), 28.3% (95% PI: 19.9%–37.6%, n = 19; Fig. 4), and 30.4% (95% PI: 18.4%–43.9%, n = 7; eFigure 2), respectively.

**Fig. 5:**
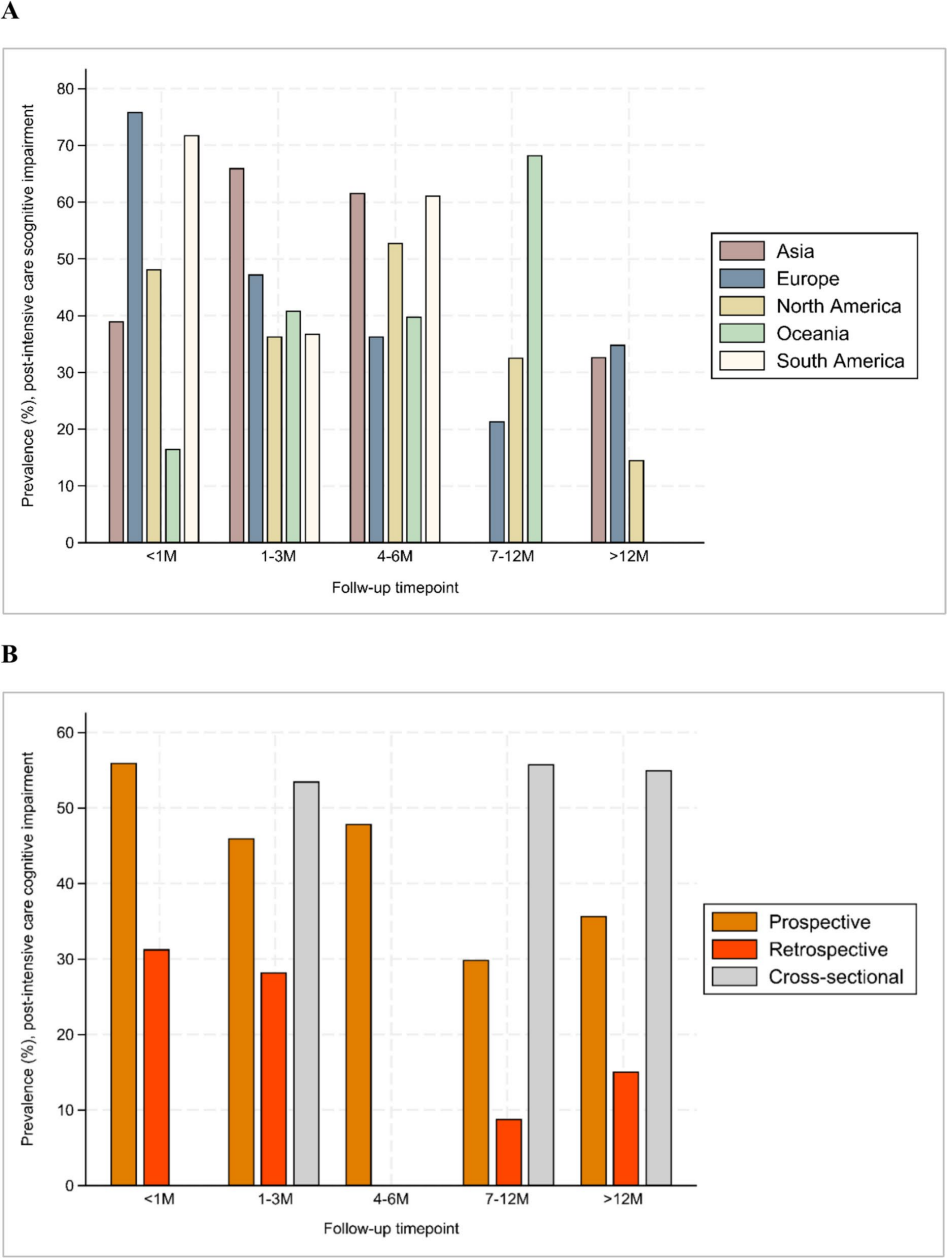
Pooled prevalence rates of post-intensive care cognitive impairment in different continents (**A**) and Study Designs (**B**) Subgroups Across all Follow-up Timepoints

Subgroup analysis showed that significant differences of the post-intensive care cognitive impairment prevalence between continents were observed at the follow-up timepoints < 1 month (*p* < 0.001), 7–12 months (*p* < 0.001), and > 12 months (*p* < 0.001). Subgroup differences were also observed between study designs the follow up timepoints < 1 month (*p* < 0.001), 1–3 month(s) (*p* = 0.01), 7–12 months (*p* < 0.001), and > 12 months (*p* < 0.001). No significant subgroup differences were observed between sample sizes after Bonferroni correction. In addition, the prevalence of post-intensive care cognitive impairment did not differ by those study population only included COVID-19 survivors at all follow-up timepoints (eFigures. 3–7). Figure 5. Summarizes the prevalence rates of post-intensive care cognitive impairment in different continents (Fig. 5A) and study designs (Fig. 5B) subgroups across all follow-up timepoints. Egger’s test revealed no small-study effects at all follow-up timepoints [< 1 month (*p* = 0.129), 1–3 month(s) (*p* = 0.588), 4–6 months (*p* = 0.936), 7–12 months (*p* = 0.070), > 12 months (*p* = 0.111)]. Leave-one-out analyses addressed identified outliers and influential studies and demonstrated that excluding single study at a time yielded similar prevalence rates of post-intensive care cognitive impairment (eFigures. 8–12). While the outlier analysis indicates that the skewed positive study did not alter the overall results, these findings should be interpreted with caution due to potential dose/effect relationships that may influence outcomes at varying doses.

## Discussion

This proportional meta-analysis was undertaken to estimate the overall prevalence of cognitive impairment among patients discharged from ICU at short-term and long-term follow-ups. Our findings indicated that the post-intensive care cognitive impairment prevalence rates can be as high as 49.8% within one months after ICU discharge. For those patients who discharged from the ICU for more than one year, the estimated prevalence was 28.3%, indicating that nearly one-third of the ICU survivors developed cognitive impairment at the long-term follow-up assessment. This study adds to the existing knowledge by providing estimates of the prevalence of cognitive impairment after intensive care at short and long-term follow-ups.

The study found that the prevalence rates of post-intensive care cognitive impairment varied at different follow-up timepoints. The high prevalence rates of post-intensive care cognitive impairment within the first three months of follow-up, suggest that cognitive impairment is a common post-ICU complication of critical illness that may need more attention during this time period. It is recommended that critical care practitioners expand their focus beyond the immediate ICU period and also consider the acute post-ICU survival period, which covers the 30 days after ICU discharge, as well as the long-term period that follows for months or even years [7, 11]. This approach would enable a more comprehensive evaluation of the outcomes and care of ICU survivors and allow for interventions to prevent or manage post-intensive care cognitive impairment. Dean and colleagues summarized the screening instruments for post-intensive care cognitive impairment and suggested that MoCA, MoCA-blind (without visual elements), and Mini-Mental State Examination (MMSE) are the commonly used for cognitive impairment screening for multiple cognitive functioning domains [9]. In our study, we also found that MoCA in all forms such as telephone MoCA, mini MoCA, and MoCA-blind were widely adopted alongside neuropsychological assessments in the included studies. Using validated screening tool is of particular important for the early detention for the post-intensive care cognitive impairment and allows the optimal intervention such as early mobilization to be implemented. Given that the risk factors of cognitive impairment have been extensively studied [11, 78, 79], it is crucial to select the most effective intervention to alleviate this condition.

As for the long-term, prolonged post-intensive care cognitive impairment, research recommended that cognitive rehabilitation showed a promising effect on cognitive function at a 3-month follow-up. Survivors who received the post-discharge cognitive rehabilitation was also demonstrated a better improvement than those who received traditional post-discharge care with only physical, occupational rehabilitation, and nursing care [80]. In addition, aerobic exercise is also found to be effective on improving prolonged cognitive impairment [81]. However, although there are various interventions accessible for post-intensive care cognitive impairment, its incidence rate remains high. Consequently, it is imperative to address this issue by conducting research to better comprehend the underlying mechanisms of post-intensive care cognitive impairment and to identify effective approaches for prevention and management. Moreover, healthcare practitioners should be educated on the significance of recognizing and treating post-intensive care cognitive impairment as well as the interventions available. This will aid in improving outcomes for ICU survivors and reducing the burden of post-intensive care syndrome on patients, families, and healthcare systems.

Given that the report from a stakeholders’ conference on improving long-term outcomes after discharge from ICU has suggested that additional investigations with larger samples and other geographic settings are needed [5], we performed subgroup analyses to detect the prevalence rates in different geographical locations. In our subgroup analysis, we found that the predefined subgroups representing different geographical locations (continents: Asia, Europe, North America, Oceania, and South America) showed significant differences in the prevalence of post-intensive care cognitive impairment at the follow-up time points of < 1 month, 7–12 months, and > 12 months. These findings contribute to our understanding of the generalizability of epidemiological evidence on post-intensive care cognitive impairment.

Our subgroup analysis also indicated that there is an absence of significant differences in prevalence rates between studies that only included COVID-19 survivors suggests that the prevalence of post-intensive care cognitive impairment in COVID-19 survivors is similar to that of ICU survivors with other types of critical illness. The COVID-19 pandemic resulted in a significant increase in ICU admission rates, with early studies reporting rates of 32% of all COVID-19 patients. This has led to a larger number of ICU survivors who may be in need of care [82]. At present, we are able to estimate the prevalence of long-term cognitive impairment in this COVID-19 survivor population. This is the first study to compare post-intensive care cognitive impairment prevalence between COVID-19 survivors alone and all ICU survivors. The finding suggests that COVID-19 patients may be at a similar risk of developing short-term and prolonged cognitive impairment as other ICU survivors. The field of neuropsychiatry for ICU survivors is still emerging and is even more critical in the post COVID-19 era.

## Strengths and limitations

This review’s strength lies in its comprehensive literature search utilized six databases, which resulted in the inclusion of a large number of studies from diverse geographic regions for meta-analyses. Validated appraisal tool was utilized to assess the methodological quality of included studies, and none of the studies included in this meta-analysis was found to have a high risk of bias. Another advantage of this review is the rigorous selection criteria, which excluded research that used non-validated measures, such as self-developed surveys.

Although this review has several strengths, it also has certain limitations that must be taken into account. First, the significant heterogeneity was observed in our pooled analyses, which suggests that the results should be interpreted with caution. This issue is not unique to our study, as other proportional meta-analyses investigating the prevalence of a particular condition have also reported marked heterogeneity, which is reflected in their elevated *I*^*2*^ values [83]. In proportional meta-analyses, high *I*^*2*^ values are expected and may not represent important between-study heterogeneity [83]. Second, despite prevalence in subgroups were compared at the regional level, studies were mainly conducted in North America and Europe (predominately the United States), whereas Oceania and South America populations were underrepresented. The generalizability of the prevalence estimates to other regions may be constrained due to certain limitations of this review. Third, when multiple instruments were used to assess cognitive function, we selected prevalence data from the most frequently used instrument across the studies which may cause the selection bias. Fourth, a notable limitation across studies is the lack of consideration for the factors influencing patient improvement following initial assessments at follow-up. Specifically, it remains uncertain whether enhancements in patient outcomes result from the mere passage of time or from interventions provided by various specialists, such as psychiatrists, physiotherapists, speech therapists, neuropsychologists, and occupational therapists. These professionals can significantly contribute to the recovery of neurocognitive functions that may be impaired due to prolonged ICU hospitalizations. This gap highlights the necessity for more comprehensive research methodologies that incorporate these variables into evaluations of patient progress post-discharge. Lastly, the exclusion of non-English articles could have led to the exclusion of pertinent studies.

## Conclusion

The prevalence rates of post-intensive care cognitive impairment differed at different follow-up timepoints. The rates were highest within the first three months of follow-up, with a pooled prevalence of 49.8% at less than one month, 45.1% at one to three months, and 47.9% at three to six months. The prevalence rates decreased at longer follow-up periods, with 28.3% at 7–12 months and 30.4% at more than 12 months. Subgroup analysis revealed significant differences in the prevalence rates of post-intensive care cognitive impairment between continents and study designs at certain follow-up timepoints. However, there were no significant differences in prevalence rates between sample sizes. The study did not find any significant differences in prevalence rates between studies that only included COVID-19 survivors. This study highlights the need for further research to develop targeted interventions to prevent or manage cognitive impairment at short-term and long-term follow-ups.

## Supplementary Information

Additional file 1.

## Data Availability

The author confirms that all data generated or analyzed during this study are included in this published article.

## Abbreviations

ARDS: Acute respiratory distress syndrome
COVID-19: Coronavirus disease 2019
ICD: International classification of diseases
ICU: Intensive care unit
MeSH: Medical subject headings
MMSE: Mini-mental state examination
MoCA: Montreal cognitive assessment
MOOSE: Meta-analysis of observational studies in epidemiology
PRISMA: Preferred reporting items for systematic reviews and meta-analyses
PICS: Post-intensive care syndrome
SCCM: Society of critical care medicine
