# Supplementary material for: Estimated prevalence of post-intensive care cognitive impairment at short-term and long-term follow-ups: a proportional meta-analysis of observational studies
Source: Ann Intensive Care. 2025 Jan 10;15:3. doi: 10.1186/s13613-025-01429-z (PMC11723879; doi:10.1186/s13613-025-01429-z)
Supplement: Supplementary file 1 — Additional file 1. [file 13613_2025_1429_MOESM1_ESM.docx]

**Estimated prevalence of post-intensive care cognitive impairment at short-term and long-term follow-ups: A proportional meta-analysis of observational studies**

**Authors:** Mu-Hsing Ho, PhD^1^, Yi-Wei Lee, MSc^2^, Lizhen Wang, MNurs^1^

**Affiliations:** ^1^School of Nursing, LKS Faculty of Medicine, The University of Hong Kong; ^2^Sijhih Cathay General Hospital, New Taipei City, Taiwan

**Authors’ information:**

*Mu-Hsing Ho*, Assistant Professor, The University of Hong Kong

E-mail address: mhbho@hku.hk; ORCID iD: 0000-0002-9443-4082; Twitter: @muhsingho

*Yi-Wei Lee*, Nurse Practitioner, Sijhih Cathay General Hospital

E-mail address: d51np@cgh.org.tw

*Lizhen Wang*, Research Assistant, The University of Hong Kong

E-mail address: u3608791@connect.hku.hk; ORCID iD: 0009-0002-5424-3705

**Corresponding author:** Mu-Hsing Ho

Address: 5/F, 3 Sassoon Road, Academic Building, Pokfulam, Hong Kong SAR

Phone: +852 3910 2787

**Estimated prevalence of post-intensive care cognitive impairment at short-term and long-term follow-ups: A** **proportional meta-analysis of observational studies**

**Abstract (350/350 words)**

**Objective:** Evidence of the overall estimated prevalence of post-intensive care cognitive impairment among critically ill survivors discharged from intensive care units at short-term and long-term follow-ups is lacking. This study aimed to estimate the prevalence of the post-intensive care cognitive impairment at time to <1 month, 1 to 3 month(s), 4 to 6 months, 7-12 months, and >12 months discharged from intensive care units.

**Methods:** Electronic databases including PubMed, Cochrane Library, EMBASE, CINAHL Plus, Web of Science, and PsycINFO via ProQuest were searched from inception through July 2024. Studies that reported on cognitive impairment among patients discharged from intensive care units with valid measures were included. Data extraction and risk of bias assessment were performed independently for all included studies according to the Preferred Reporting Items for Systematic Reviews and Meta-analyses reporting guidelines. Newcastle–Ottawa Scale was used to measure risk of bias. Data on cognitive impairment prevalence were pooled using a random-effects model. The primary outcome was pooled estimated proportions of prevalence of the post-intensive care cognitive impairment.

**Results:** In total, 58 studies involving 347,940 patients were included. The pooled post-intensive care cognitive impairment prevalence rates at the follow-up timepoints <1 month, 1-3 month(s), 4-6 months, 7-12 months, >12 months were 49.8% [95% Prediction Interval (PI), 39.9%–59.7%, n = 19], 45.1% (95% PI, 34.8%–55.5%, n = 23), 47.9% (95% PI, 35.9%–60.0%, n = 16), 28.3% (95% PI, 19.9%–37.6%, n = 19), and 30.4% (95% PI, 18.4%–43.9%, n = 7), respectively. Subgroup analysis showed that significant differences of the prevalence rates between continents and study designs were observed.

**Conclusions:** The prevalence rates of post-intensive care cognitive impairment differed at different follow-up timepoints. The rates were highest within the first three months of follow-up, with a pooled prevalence of 49.8% at less than one month, 45.1% at one to three months, and 47.9% at three to six months. No significant differences in prevalence rates between studies that only included coronavirus disease 2019 survivors. These fundings highlight the need for further research to develop targeted interventions to prevent or manage cognitive impairment at short-term and long-term follow-ups.

**Keywords:** cognitive impairment; critically ill survivors; post-intensive care syndrome; proportional meta-analysis; systematic review.

**Background**

The combination of higher intensive care unit (ICU) survival rates and an aging global population is likely to result in a greater need for ICU resources. With the proportion of elderly individuals in the population expected to rise to 20% by 2050, the demand for ICU care is likely to increase, highlighting the need for effective critical illness and post-intensive care syndrome (PICS) management [1, 2]. PICS affects cognitive, physical, and psychological sequelae that impact the quality of life and persist beyond hospital discharge among critically ill survivors. It can include a range of symptoms, such as muscle weakness, cognitive impairment, mental and emotional distress, as well as post-traumatic stress disorder[3]. PICS is a significant issue for ICU survivors, and there is a need for clinicians to screen for it and for policy development to address its impact [4]. The Society of Critical Care Medicine (SCCM) defined PICS as “a new or worsening impairment of cognition, mental health, or physical function after critical illness that persists beyond the acute care hospitalization [5].” PICS is considered to have a multifactorial etiology and is associated with inflammatory conditions, for example, sepsis and acute respiratory distress syndrome (ARDS). During the COVID-19 pandemic, there was a significant increase in the number of critically ill patients developing ARDS. COVID-19 infection has been shown to cause COVID-19 ARDS, which can result in patients receiving mechanical ventilation and ICU-level support. This association between COVID-19 infection and ARDS highlights the potential for COVID-19 to contribute to the development of PICS in affected patients [6].

One of the primary features of PICS is cognitive dysfunction, which can be attributed to various factors. Prolonged stays in ICUs, the need for mechanical ventilation, use of sedative and steroid medications, use of physical restraints, prolonged immobility, and underlying comorbidities are all potential contributors to PICS development. These factors can interact and exacerbate each other, leading to long-term cognitive dysfunction that may persist even after hospital discharge, underscoring the importance of addressing PICS risk factors in ICU patients [7]. Older age, have a mental illness, or neurological diseases are at a higher risk of developing this condition [8, 9]. The mechanisms of cognitive impairment in critically ill survivors are not yet fully understood due to the diversity of ICU patient populations and disease diagnoses [7]. Cognitive impairment in critically ill patients is a multifaceted issue that likely involves a combination of various risk factors, including comorbidities, genetic factors, and exposure to specific ICU-related factors [10]. Hypoxemia, the duration of hypoxemia, delirium, hypotension, glucose dysregulation, pre-existing mental health problems, inflammation, and cytokine activation are all potential contributors to ICU-related cognitive impairment [11, 12]. However, these factors and their interactions have not been fully elucidated. It is unclear whether cognitive sequelae in ICU patients can be ameliorated [10, 11]. Currently, there is no summary evidence of the overall estimated prevalence of cognitive impairment among critically ill survivors discharged from ICU at short-term and long-term follow-ups. A review of 19 studies reported a wide range of cognitive impairment in 4-62% of patients during post-ICU periods ranging from 2 to 156 months [10]. The prevalence rates varied greatly, and the review did not explicitly highlight a specific time to follow-up, as it included studies that evaluated patients more than two months after ICU discharge. The prevalence of short-term follow-up (less than two months) remains unknown. Given that there is an increasing number of the prospective studies investigated the post-intensive care cognitive impairment in a longitudinal follow-up time period. This meta-analysis aimed to estimate the overall prevalence of post-intensive care cognitive impairment among critically ill survivors discharged from the ICU, considering regional differences across studies, and individual characteristics.

**Methods**

The guidelines including Meta-Analysis of Observational Studies in Epidemiology (MOOSE)[13] and Preferred Reporting Items for Systematic reviews and Meta-Analyses (PRISMA) [14] were followed in this systematic review and proportional meta-analysis (eTable 1). The review registration number on PROSPERO is CRD42024570155.

**Search strategy**

A comprehensive search was conducted across multiple databases such as PubMed, Cochrane Library, CINAHL Plus, EMBASE, PsycINFO via ProQuest and Web of Science. The search was performed using MeSH terms and relevant keywords such as cognitive dysfunction and intensive care units, and included all articles from the inception of the databases up to August 2024. The complete search strategy is outlined in eTable 2. Additionally, a manual citation chasing was performed to identify any relevant articles that were eligible.

**Eligible criteria**

Inclusion criteria for the studies were as follows: 1) observational studies with retrospective and prospective cohort, or cross-sectional design reporting on the prevalence of cognitive impairment; 2) patient population consisting of ICU survivors who were admitted to and discharged from ICU; and 3) identification of cognitive impairment using validated measures and available to define cognitive impairment or diagnosis by physicians. In the case of multiple articles utilizing data from the same patient source, the study with the most comprehensive information or the largest sample size was included to avoid overcounting the prevalence of post-intensive care cognitive impairment. Exclusion criteria were as follows: 1) editorials, reviews, letters to the editor, discussion paper, or conference abstracts with insufficient prevalence data; 2) failure to provide an operational definition of cognitive impairment; 3) observation of cognitive impairment prevalence in ICU without post-ICU follow-up observations; or 4) publication not in English language.

**Study selection and data extraction**

The title and abstract of all records identified in the literature search were independently screened by five reviewers, including MHH, YWL, and three research assistants with experience in conducting systematic reviews. Remaining articles’ full texts were independently reviewed by the same five reviewers. Each article was assessed by at least three reviewers independently. To ensure consistency and accuracy, we created a data extraction sheet in a standardized format for reviewers to record study details such as publication information (first author, year of publication, and sample size), country/region, study design, where the study was conducted, COVID-19 patients involved, illness severity scoring systems including acute physiology and chronic health evaluation (APACHE), simplified acute physiology score (SAPS), sequential organ failure assessment (SOFA), and cognitive function measures, as well as sample characteristics including mean age, percentage in male, and the number of cases developed post-intensive care cognitive impairment. The information was extracted independently and reviewed for consistency and accuracy. The research team reach a consensus through discussion if there were any discrepancies.

**Risk of bias assessment**

Two reviewers (MHH and YWL) assessed the risk of bias of included studies using the Newcastle-Ottawa Scale [15]. This scale comprises three sections, which assess sample selection, comparability of study groups, and exposure/outcome assessment. The scale includes questions about the representativeness of the sample, adjustment for confounders, appropriateness of exposure/outcome measures, and adequacy of follow-up or participant response rates to determine the risk of bias in a study. Assessment criteria were detailed in eTable3. The final decision of studies inclusion and exclusion was made through discussion with the research team.

**Data synthesis and analysis**

The Freeman–Tukey double arcsine transformation of proportions was adopted to pool prevalence estimates in this proportional meta-analysis [16, 17]. The follow-up timepoints for the cognitive function assessments were varied, and several studies have reported multiple follow-ups across time, thus the follow-up timepoints were categorized into five timepoints [<1 month, 1-3 month(s), 4-6 months, 7-12 months, >12 months] for understanding the prevalence of post-intensive care cognitive impairment in a short-term and long-term period after ICU discharge. Given the diversity of patients undergoing various treatment procedures and diagnoses, as well as the inherent heterogeneity of prevalence data, we anticipated substantial between-study heterogeneity. Therefore, this proportional meta-analysis was performed using a random-effects model [18]. In situations where multiple assessment tools were used to measure cognitive impairment, prevalence data were obtained for meta-analysis from the most frequently used assessment tools. The pooled prevalence estimates are expressed as percentages, along with 95% prediction intervals (PIs).

To assess statistical heterogeneity, we used Cochran’s *Q* test and *I^2^* statistics, where *I^2^* values of 75% suggested high heterogeneity [19]. The predefined subgroups, including the geographical location of the study (continents: Asia, Europe, North America, Oceania, and South America), study design (prospective, retrospective, or cross-sectional), sample size (<100 or ≥100), and studies focus on COVID-19 survivors only were used to explore potential study-level factors contributing to heterogeneity in the prevalence of post-intensive care cognitive impairment. To reduce the family-wise type I error resulting from multiple comparisons within subgroups, we applied Bonferroni correction to the significance level.

To evaluate the robustness of the pooled prevalence estimates, we conducted sensitivity analyses by excluding studies. We also performed leave-one-out analyses to determine the impact of outliers. Egger’s test was used to evaluate small-study effects. A significant level of 0.05 was set. All statistical analyses were conducted using Stata 18 software.

**Results**

**Search Strategy and Study Characteristics**

The initial search produced a total of 91,93 records from all databases, which 7,410 records were retained following the elimination of duplicates. From these, 58 studies that were deemed eligible according to the pre-specified inclusion criteria, and had been published between 2006 and 2024, were ultimately selected for inclusion in this review (Figure 1). Of all studies, 75.9% (n = 44) used a prospective cohort design [20-63], 17.2% (n = 10) used a retrospective cohort design [64-73], and 6.9% used a cross-sectional design (n = 4) [74-77] for reporting the quantitative results. Most studies were conducted in North America (39.8%, n = 23), followed by Europe (36.2%, n = 21), Asia (17.2%, n = 10), Oceania (3.4%, n = 2), and South America (3.4%, n = 2). The most common measurement for cognitive function was the Montreal Cognitive Assessment (MoCA) and a total of 22 studies (37.9%) adopted MoCA to identify post-intensive care cognitive impairment. A total of 347,940 patients were included, with the mean age ranging from 42.7 to 83.5 years, and 38.5–84.3% of these patients were men. Table 1 shows the key characteristics of the included studies.

**Risk of Bias Assessment**

The risk of bias assessment result is summarized in eTable 3. The methodological quality of all studies deemed satisfactory, and no study was excluded due to the risk of bias assessment. Most studies were rated as having good quality, the variations in scores were primarily attributed to the absence or ambiguity of adjustments made for confounding factors.

**Prevalence of Post-Intensive Care Cognitive Impairment**

The pooled prevalence rates of post-intensive care cognitive impairment at the follow-up timepoints <1 month, 1-3 month(s), 4-6 months, 7-12 months, >12 months were 49.8% (95% PI: 39.9%–59.7%, n = 19; Figure 2), 45.1% (95% PI: 34.8%–55.5%, n = 23; Figure 3), 47.9% (95% PI: 35.9%–60.0%, n = 16; eFigure 1), 28.3% (95% PI: 19.9%–37.6%, n = 19; Figure 4), and 30.4% (95% PI: 18.4%–43.9%, n = 7; eFigure 2), respectively.

Subgroup analysis showed that significant differences of the post-intensive care cognitive impairment prevalence between continents were observed at the follow-up timepoints <1 month (*p* <0.001), 7-12 months (*p* <0.001), and >12 months (*p* <0.001). Subgroup differences were also observed between study designs the follow up timepoints <1 month (*p* <0.001), 1-3 month(s) (*p* = 0.01), 7-12 months (*p* <0.001), and >12 months (*p* <0.001). No significant subgroup differences were observed between sample sizes after Bonferroni correction. In addition, the prevalence of post-intensive care cognitive impairment did not differ by those study population only included COVID-19 survivors at all follow-up timepoints (eFigures. 3-7). Figure 5. summarizes the prevalence rates of post-intensive care cognitive impairment in different continents (Figure 5A) and study designs (Figure 5B) subgroups across all follow-up timepoints. Egger’s test revealed no small-study effects at all follow-up timepoints [<1 month (*p* = 0.129), 1-3 month(s) (*p* = 0.588), 4-6 months (*p* = 0.936), 7-12 months (*p* = 0.070), >12 months (*p* = 0.111)]. Leave-one-out analyses addressed identified outliers and influential studies and demonstrated that excluding single study at a time yielded similar prevalence rates of post-intensive care cognitive impairment (eFigures. 8-12). While the outlier analysis indicates that the skewed positive study did not alter the overall results, these findings should be interpreted with caution due to potential dose/effect relationships that may influence outcomes at varying doses.

**Discussion**

This proportional meta-analysis was undertaken to estimate the overall prevalence of cognitive impairment among patients discharged from ICU at short-term and long-term follow-ups. Our findings indicated that the post-intensive care cognitive impairment prevalence rates can be as high as 49.8% within one months after ICU discharge. For those patients who discharged from the ICU for more than one year, the estimated prevalence was 28.3%, indicating that nearly one-third of the ICU survivors developed cognitive impairment at the long-term follow-up assessment. This study adds to the existing knowledge by providing estimates of the prevalence of cognitive impairment after intensive care at short and long-term follow-ups.

The study found that the prevalence rates of post-intensive care cognitive impairment varied at different follow-up timepoints. The high prevalence rates of post-intensive care cognitive impairment within the first three months of follow-up, suggest that cognitive impairment is a common post-ICU complication of critical illness that may need more attention during this time period. It is recommended that critical care practitioners expand their focus beyond the immediate ICU period and also consider the acute post-ICU survival period, which covers the 30 days after ICU discharge, as well as the long-term period that follows for months or even years [7, 11]. This approach would enable a more comprehensive evaluation of the outcomes and care of ICU survivors and allow for interventions to prevent or manage post-intensive care cognitive impairment. Dean and colleagues summarized the screening instruments for post-intensive care cognitive impairment and suggested that MoCA, MoCA-blind (without visual elements), and Mini-Mental State Examination (MMSE) are the commonly used for cognitive impairment screening for multiple cognitive functioning domains [9]. In our study, we also found that MoCA in all forms such as telephone MoCA, mini MoCA, and MoCA-blind were widely adopted alongside neuropsychological assessments in the included studies. Using validated screening tool is of particular important for the early detention for the post-intensive care cognitive impairment and allows the optimal intervention such as early mobilization to be implemented. Given that the risk factors of cognitive impairment have been extensively studied [11, 78, 79], it is crucial to select the most effective intervention to alleviate this condition.

As for the long-term, prolonged post-intensive care cognitive impairment, research recommended that cognitive rehabilitation showed a promising effect on cognitive function at a 3-month follow-up. Survivors who received the post-discharge cognitive rehabilitation was also demonstrated a better improvement than those who received traditional post-discharge care with only physical, occupational rehabilitation, and nursing care [80]. In addition, aerobic exercise is also found to be effective on improving prolonged cognitive impairment [81]. However, although there are various interventions accessible for post-intensive care cognitive impairment, its incidence rate remains high. Consequently, it is imperative to address this issue by conducting research to better comprehend the underlying mechanisms of post-intensive care cognitive impairment and to identify effective approaches for prevention and management. Moreover, healthcare practitioners should be educated on the significance of recognizing and treating post-intensive care cognitive impairment as well as the interventions available. This will aid in improving outcomes for ICU survivors and reducing the burden of post-intensive care syndrome on patients, families, and healthcare systems.

Given that the report from a stakeholders’ conference on improving long-term outcomes after discharge from ICU has suggested that additional investigations with larger samples and other geographic settings are needed [5], we performed subgroup analyses to detect the prevalence rates in different geographical locations. In our subgroup analysis, we found that the predefined subgroups representing different geographical locations (continents: Asia, Europe, North America, Oceania, and South America) showed significant differences in the prevalence of post-intensive care cognitive impairment at the follow-up time points of <1 month, 7-12 months, and >12 months. These findings contribute to our understanding of the generalizability of epidemiological evidence on post-intensive care cognitive impairment.

Our subgroup analysis also indicated that there is an absence of significant differences in prevalence rates between studies that only included COVID-19 survivors suggests that the prevalence of post-intensive care cognitive impairment in COVID-19 survivors is similar to that of ICU survivors with other types of critical illness. The COVID-19 pandemic resulted in a significant increase in ICU admission rates, with early studies reporting rates of 32% of all COVID-19 patients. This has led to a larger number of ICU survivors who may be in need of care [82]. At present, we are able to estimate the prevalence of long-term cognitive impairment in this COVID-19 survivor population. This is the first study to compare post-intensive care cognitive impairment prevalence between COVID-19 survivors alone and all ICU survivors. The finding suggests that COVID-19 patients may be at a similar risk of developing short-term and prolonged cognitive impairment as other ICU survivors. The field of neuropsychiatry for ICU survivors is still emerging and is even more critical in the post COVID-19 era.

**Strengths and Limitations**

This review’s strength lies in its comprehensive literature search utilized six databases, which resulted in the inclusion of a large number of studies from diverse geographic regions for meta-analyses. Validated appraisal tool was utilized to assess the methodological quality of included studies, and none of the studies included in this meta-analysis was found to have a high risk of bias. Another advantage of this review is the rigorous selection criteria, which excluded research that used non-validated measures, such as self-developed surveys.

Although this review has several strengths, it also has certain limitations that must be taken into account. First, the significant heterogeneity was observed in our pooled analyses, which suggests that the results should be interpreted with caution. This issue is not unique to our study, as other proportional meta-analyses investigating the prevalence of a particular condition have also reported marked heterogeneity, which is reflected in their elevated *I^2^* values [83]. In proportional meta-analyses, high *I^2^* values are expected and may not represent important between-study heterogeneity [83]. Second, despite prevalence in subgroups were compared at the regional level, studies were mainly conducted in North America and Europe (predominately the United States), whereas Oceania and South America populations were underrepresented. The generalizability of the prevalence estimates to other regions may be constrained due to certain limitations of this review. Third, when multiple instruments were used to assess cognitive function, we selected prevalence data from the most frequently used instrument across the studies which may cause the selection bias. Fourth, a notable limitation across studies is the lack of consideration for the factors influencing patient improvement following initial assessments at follow-up. Specifically, it remains uncertain whether enhancements in patient outcomes result from the mere passage of time or from interventions provided by various specialists, such as psychiatrists, physiotherapists, speech therapists, neuropsychologists, and occupational therapists. These professionals can significantly contribute to the recovery of neurocognitive functions that may be impaired due to prolonged ICU hospitalizations. This gap highlights the necessity for more comprehensive research methodologies that incorporate these variables into evaluations of patient progress post-discharge. Lastly, the exclusion of non-English articles could have led to the exclusion of pertinent studies.

**Conclusion**

The prevalence rates of post-intensive care cognitive impairment differed at different follow-up timepoints. The rates were highest within the first three months of follow-up, with a pooled prevalence of 49.8% at less than one month, 45.1% at one to three months, and 47.9% at three to six months. The prevalence rates decreased at longer follow-up periods, with 28.3% at 7-12 months and 30.4% at more than 12 months. Subgroup analysis revealed significant differences in the prevalence rates of post-intensive care cognitive impairment between continents and study designs at certain follow-up timepoints. However, there were no significant differences in prevalence rates between sample sizes. The study did not find any significant differences in prevalence rates between studies that only included COVID-19 survivors. This study highlights the need for further research to develop targeted interventions to prevent or manage cognitive impairment at short-term and long-term follow-ups.

**List of abbreviations:**

***ARDS:*** Acute respiratory distress syndrome

***COVID-19:*** Coronavirus disease 2019

***ICD:*** International Classification of Diseases

***ICU:*** Intensive care unit

***MeSH:*** Medical subject headings

***MMSE:*** Mini-Mental State Examination

***MoCA:*** Montreal Cognitive Assessment

***MOOSE:*** Meta-Analysis of Observational Studies in Epidemiology

***PRISMA:*** Preferred Reporting Items for Systematic reviews and Meta-Analyses

***PICS:*** Post-intensive care syndrome

***SCCM:*** Society of Critical Care Medicine

**Declarations**

**Ethics approval:** Not applicable as this is a systematic review and meta-analysis.

**Consent for publication:** Not applicable.

**Availability of data and material:** The author confirms that all data generated or analyzed during this study are included in this published article.

**Competing interests:** Dr Mu-Hsing Ho reported receiving grant funding from the Health and Medical Research Fund (HMRF), Health Bureau, Hong Kong SAR and HMRF Research Fellowship Scheme, Health Bureau, Hong Kong SAR during the conduct of the study. No other disclosures were reported.

**Acknowledgements:** Not applicable.

**Funding:** No funds, grants, or other support was received.

**Authors’ contributions:** All authors commented on previous versions of the manuscript. All authors read and approved the final manuscript. Dr Mu-Hsing Ho had full access to all of the data in the study and take responsibility for the integrity of the data and the accuracy of the data analysis.

*Concept and design:* Mu-Hsing Ho, Yi-Wei Lee.

*Acquisition, analysis, or interpretation of data:* Mu-Hsing Ho, Lizhen Wang.

*Drafting of the manuscript:* Mu-Hsing Ho.

*Critical review of the manuscript for important intellectual content:* Mu-Hsing Ho, Yi-Wei Lee.

*Statistical analysis:* Mu-Hsing Ho.

*Administrative, technical, or material support:* Mu-Hsing Ho, Lizhen Wang, Yi-Wei Lee.

*Supervision:* Mu-Hsing Ho.

**References**

1. Akinosoglou K, Schinas G, Almyroudi MP, Gogos C, Dimopoulos G: The impact of age on intensive care. *Ageing Res Rev* 2023, 84:101832.

2. Jackson JC, Ely EW: Cognitive impairment after critical illness: etiologies, risk factors, and future directions. *Semin Respir Crit Care Med* 2013, 34(2):216-222.

3. Hiser SL, Fatima A, Ali M, Needham DM: Post-intensive care syndrome (PICS): recent updates. *J Intensive Care* 2023, 11(1):23.

4. Peach BC, Valenti M, Sole ML: A Call for the World Health Organization to Create International Classification of Disease Diagnostic Codes for Post-Intensive Care Syndrome in the Age of COVID-19. *World Med Health Policy* 2021, 13(2):373-382.

5. Needham DM, Davidson J, Cohen H, Hopkins RO, Weinert C, Wunsch H, Zawistowski C, Bemis-Dougherty A, Berney SC, Bienvenu OJ *et al*: Improving long-term outcomes after discharge from intensive care unit: report from a stakeholders' conference. *Crit Care Med* 2012, 40(2):502-509.

6. Pandharipande P, Williams Roberson S, Harrison FE, Wilson JE, Bastarache JA, Ely EW: Mitigating neurological, cognitive, and psychiatric sequelae of COVID-19-related critical illness. *Lancet Respir Med* 2023, 11(8):726-738.

7. Skrobik Y, Hopkins RO: Post-intensive care cognitive impairment: questions in mind? *Intensive Care Med* 2013, 39(3):524-527.

8. Jackson JC, Gordon SM, Ely EW, Burger C, Hopkins RO: Research issues in the evaluation of cognitive impairment in intensive care unit survivors. *Intensive Care Med* 2004, 30(11):2009-2016.

9. Dean EA, Biehl M, Bash K, Weleff J, Pozuelo L: Neuropsychiatric assessment and management of the ICU survivor. *Cleve Clin J Med* 2021, 88(12):669-679.

10. Wolters AE, Slooter AJ, van der Kooi AW, van Dijk D: Cognitive impairment after intensive care unit admission: a systematic review. *Intensive Care Med* 2013, 39(3):376-386.

11. Lee M, Kang J, Jeong YJ: Risk factors for post-intensive care syndrome: A systematic review and meta-analysis. *Aust Crit Care* 2020, 33(3):287-294.

12. Mikkelsen ME, Still M, Anderson BJ, Bienvenu OJ, Brodsky MB, Brummel N, Butcher B, Clay AS, Felt H, Ferrante LE *et al*: Society of Critical Care Medicine's International Consensus Conference on Prediction and Identification of Long-Term Impairments After Critical Illness. *Crit Care Med* 2020, 48(11):1670-1679.

13. Stroup DF, Berlin JA, Morton SC, Olkin I, Williamson GD, Rennie D, Moher D, Becker BJ, Sipe TA, Thacker SB: Meta-analysis of observational studies in epidemiology: a proposal for reporting. Meta-analysis Of Observational Studies in Epidemiology (MOOSE) group. *Jama* 2000, 283(15):2008-2012.

14. Page MJ, McKenzie JE, Bossuyt PM, Boutron I, Hoffmann TC, Mulrow CD, Shamseer L, Tetzlaff JM, Akl EA, Brennan SE *et al*: The PRISMA 2020 statement: an updated guideline for reporting systematic reviews. *Bmj* 2021, 372:n71.

15. Wells GA, Shea B, O’Connell D, Peterson J, Welch V, Losos M, Tugwell P: The Newcastle-Ottawa Scale (NOS) for assessing the quality of nonrandomised studies in meta-analyses. 2000.

16. Field AP, Gillett R: How to do a meta-analysis. *Br J Math Stat Psychol* 2010, 63(Pt 3):665-694.

17. Freeman MF, Tukey JW: Transformations related to the angular and the square root. *The annals of mathematical statistics* 1950:607-611.

18. Borenstein M, Hedges LV, Higgins JP, Rothstein HR: A basic introduction to fixed-effect and random-effects models for meta-analysis. *Res Synth Methods* 2010, 1(2):97-111.

19. Higgins JP, Thompson SG: Quantifying heterogeneity in a meta-analysis. *Stat Med* 2002, 21(11):1539-1558.

20. Balasubramanian V, Suri JC, Ish P, Gupta N, Behera D, Gupta P, Chakrabarti S: Neurocognitive and Quality-of-life Outcomes Following Intensive Care Admission: A Prospective 6-month Follow-up Study. *Indian J Crit Care Med* 2020, 24(10):932-937.

21. Baldwin MR, Pollack LR, Friedman RA, Norris SP, Javaid A, O'Donnell MR, Cummings MJ, Needham DM, Colantuoni E, Maurer MS *et al*: Frailty subtypes and recovery in older survivors of acute respiratory failure: a pilot study. *Thorax* 2021, 76(4):350-359.

22. Bark L, Larsson IM, Wallin E, Simrén J, Zetterberg H, Lipcsey M, Frithiof R, Rostami E, Hultström M: Central nervous system biomarkers GFAp and NfL associate with post-acute cognitive impairment and fatigue following critical COVID-19. *Sci Rep* 2023, 13(1):13144.

23. Bottom-Tanzer SF, Poyant JO, Louzada MT, Abela D, Boudouvas A, Poon E, Power L, Kim WC, Hojman HM, Bugaev N *et al*: Longitudinal study evaluating post-ICU syndrome differences between acute care surgery and trauma SICU survivors. *J Trauma Acute Care Surg* 2023, 95(6):893-898.

24. Brück E, Larsson JW, Lasselin J, Bottai M, Hirvikoski T, Sundman E, Eberhardson M, Sackey P, Olofsson PS: Lack of clinically relevant correlation between subjective and objective cognitive function in ICU survivors: a prospective 12-month follow-up study. *Crit Care* 2019, 23(1):253.

25. Brück E, Schandl A, Bottai M, Sackey P: The impact of sepsis, delirium, and psychological distress on self-rated cognitive function in ICU survivors-a prospective cohort study. *J Intensive Care* 2018, 6:2.

26. Bulic D, Bennett M, Georgousopoulou EN, Shehabi Y, Pham T, Looi JCL, van Haren FMP: Cognitive and psychosocial outcomes of mechanically ventilated intensive care patients with and without delirium. *Ann Intensive Care* 2020, 10(1):104.

27. Carenzo L, Zini L, Mercalli C, Stomeo N, Milani A, Amato K, Gatti R, Costantini E, Aceto R, Protti A *et al*: Health related quality of life, physical function, and cognitive performance in mechanically ventilated COVID-19 patients: A long term follow-up study. *J Crit Care* 2024, 82:154773.

28. Castro-Avila A, Merino-Osorio C, González-Seguel F, Camus-Molina A, Muñoz-Muñoz F, Leppe J: Six-month post-intensive care outcomes during high and low bed occupancy due to the COVID-19 pandemic: A multicenter prospective cohort study. *PLoS One* 2023, 18(11):e0294631.

29. Costas-Carrera A, Sánchez-Rodríguez MM, Ojeda A, Rodríguez-Rey MA, Martín-Villalba I, Primé-Tous M, Valdesoiro-Pulido F, Segú X, Borras R, Clougher D *et al*: Neuropsychological functioning and its correlates at 1 year follow-up of severe COVID-19. *Psychogeriatrics* 2024, 24(4):765-777.

30. De Tanti A, Conforti J, Bruni S, De Gaetano K, Cappalli A, Basagni B, Bertoni D, Saviola D: Cognitive and psychological outcomes and follow-up in severely affected COVID-19 survivors admitted to a rehabilitation hospital. *Neurol Sci* 2023, 44(5):1481-1489.

31. Dubin R, Veith JM, Grippi MA, McPeake J, Harhay MO, Mikkelsen ME: Functional Outcomes, Goals, and Goal Attainment among Chronically Critically Ill Long-Term Acute Care Hospital Patients. *Ann Am Thorac Soc* 2021, 18(12):2041-2048.

32. Duggan MC, Wang L, Wilson JE, Dittus RS, Ely EW, Jackson JC: The relationship between executive dysfunction, depression, and mental health-related quality of life in survivors of critical illness: Results from the BRAIN-ICU investigation. *J Crit Care* 2017, 37:72-79.

33. Fagerberg SK, Kruse M, Olesen TSW, Andersen H, Klostergaard K, Leutscher PDC: Assessment of neuropsychiatric manifestations in a cohort of intensive care unit survivors: A proof of concept study. *J Intensive Care Soc* 2023, 24(4):442-445.

34. Fernández-Gonzalo S, Navarra-Ventura G, Bacardit N, Gomà Fernández G, de Haro C, Subirà C, López-Aguilar J, Magrans R, Sarlabous L, Aquino Esperanza J *et al*: Cognitive phenotypes 1 month after ICU discharge in mechanically ventilated patients: a prospective observational cohort study. *Crit Care* 2020, 24(1):618.

35. Ferrante LE, Murphy TE, Gahbauer EA, Leo-Summers LS, Pisani MA, Gill TM: Pre-Intensive Care Unit Cognitive Status, Subsequent Disability, and New Nursing Home Admission among Critically Ill Older Adults. *Ann Am Thorac Soc* 2018, 15(5):622-629.

36. Fjone KS, Stubberud J, Buanes EA, Hagen M, Laake JH, Hofsø K: Objective and subjective cognitive status after intensive care unit treatment for COVID-19. *Brain Behav Immun Health* 2024, 38:100786.

37. Geense WW, Zegers M, Peters MAA, Ewalds E, Simons KS, Vermeulen H, van der Hoeven JG, van den Boogaard M: New Physical, Mental, and Cognitive Problems 1 Year after ICU Admission: A Prospective Multicenter Study. *Am J Respir Crit Care Med* 2021, 203(12):1512-1521.

38. Godoy-González M, Navarra-Ventura G, Gomà G, de Haro C, Espinal C, Fortià C, Ridao N, Miguel Rebanal N, Oliveras-Furriols L, Subirà C *et al*: Objective and subjective cognition in survivors of COVID-19 one year after ICU discharge: the role of demographic, clinical, and emotional factors. *Crit Care* 2023, 27(1):188.

39. Habib S, Khan A, Afridi MI, Saeed A, Jan AF, Amjad N: Frequency and predictors of cognitive decline in patients undergoing coronary artery bypass graft surgery. *J Coll Physicians Surg Pak* 2014, 24(8):543-548.

40. Haddad DN, Mart MF, Wang L, Lindsell CJ, Raman R, Nordness MF, Sharp KW, Pandharipande PP, Girard TD, Ely EW *et al*: Socioeconomic Factors and Intensive Care Unit-Related Cognitive Impairment. *Ann Surg* 2020, 272(4):596-602.

41. Hatakeyama J, Inoue S, Liu K, Yamakawa K, Nishida T, Ohshimo S, Hashimoto S, Kanda N, Maruyama S, Ogata Y *et al*: Prevalence and Risk Factor Analysis of Post-Intensive Care Syndrome in Patients with COVID-19 Requiring Mechanical Ventilation: A Multicenter Prospective Observational Study. *J Clin Med* 2022, 11(19).

42. Jackson JC, Archer KR, Bauer R, Abraham CM, Song Y, Greevey R, Guillamondegui O, Ely EW, Obremskey W: A prospective investigation of long-term cognitive impairment and psychological distress in moderately versus severely injured trauma intensive care unit survivors without intracranial hemorrhage. *J Trauma* 2011, 71(4):860-866.

43. Jaquet P, Legouy C, Le Fevre L, Grinea A, Sinnah F, Franchineau G, Patrier J, Marzouk M, Wicky PH, Alexis Geoffroy P *et al*: Neurologic Outcomes of Survivors of COVID-19-Associated Acute Respiratory Distress Syndrome Requiring Intubation. *Crit Care Med* 2022, 50(8):e674-e682.

44. Jones C, Griffiths RD, Slater T, Benjamin KS, Wilson S: Significant cognitive dysfunction in non-delirious patients identified during and persisting following critical illness. *Intensive Care Med* 2006, 32(6):923-926.

45. Kang J, Lee MH: Incidence rate and risk factors for post-intensive care syndrome subtypes among critical care survivors three months after discharge: A prospective cohort study. *Intensive Crit Care Nurs* 2024, 81:103605.

46. Karnatovskaia LV, Schulte PJ, Philbrick KL, Johnson MM, Anderson BK, Gajic O, Clark MM: Psychocognitive sequelae of critical illness and correlation with  months follow up. *J Crit Care* 2019, 52:166-171.

47. Kawakami D, Fujitani S, Morimoto T, Dote H, Takita M, Takaba A, Hino M, Nakamura M, Irie H, Adachi T *et al*: Prevalence of post-intensive care syndrome among Japanese intensive care unit patients: a prospective, multicenter, observational J-PICS study. *Crit Care* 2021, 25(1):69.

48. Klinkhammer S, Horn J, Duits AA, Visser-Meily JMA, Verwijk E, Slooter AJC, Postma AA, van Heugten CM: Neurological and (neuro)psychological sequelae in intensive care and general ward COVID-19 survivors. *Eur J Neurol* 2023, 30(7):1880-1890.

49. Kosilek RP, Schmidt K, Baumeister SE, Gensichen J: Frequency and risk factors of post-intensive care syndrome components in a multicenter randomized controlled trial of German sepsis survivors. *J Crit Care* 2021, 65:268-273.

50. Mart MF, Semler MW, Jenkins CA, Wang G, Casey JD, Ely EW, Jackson JC, Kiehl AL, Bryant PT, Pugh SK *et al*: Oxygen-Saturation Targets and Cognitive and Functional Outcomes in Mechanically Ventilated Adults. *Am J Respir Crit Care Med* 2024, 209(7):861-870.

51. Martínez E, Aguilera C, Márquez D, Ziegler G, Plumet J, Tschopp L, Cominotti C, Sturzenegger V, Cimino C, Escobar H *et al*: Post intensive care syndrome in survivors of COVID-19 who required mechanical ventilation during the third wave of the pandemic: A prospective study. *Heart Lung* 2023, 62:72-80.

52. Mimenza-Alvarado AJ, Ambrosio-Palma A, Aguilar-Navarro SG: Cognitive function in Mexican older adults 6-months after recovering from SARS-CoV-2 infection. *Rev Esp Geriatr Gerontol* 2024, 59(5):101479.

53. Mitchell ML, Shum DHK, Mihala G, Murfield JE, Aitken LM: Long-term cognitive impairment and delirium in intensive care: A prospective cohort study. *Aust Crit Care* 2018, 31(4):204-211.

54. Nordness MF, Bipin Patel M, Erickson CR, Kiehl A, Jackson JC, Raman R, Pandharipande PP, Ely EW, Wilson JE: Depression predicts long-term cognitive impairment in survivors of critical illness. *J Trauma Acute Care Surg* 2021, 90(1):79-86.

55. Pereira S, Cavaco S, Fernandes J, Moreira I, Almeida E, Seabra-Pereira F, Castro H, Malheiro MJ, Cardoso AF, Aragão I *et al*: Long-term psychological outcome after discharge from intensive care. *Rev Bras Ter Intensiva* 2018, 30(1):28-34.

56. Proffitt T, Menzies V, Grap MJ, Orr T, Thacker L, 2nd, Ameringer S: Cognitive Impairment, Physical Impairment, and Psychological Symptoms in Intensive Care Unit Survivors. *Am J Crit Care* 2023, 32(6):410-420.

57. Sevin CM, Bloom SL, Jackson JC, Wang L, Ely EW, Stollings JL: Comprehensive care of ICU survivors: Development and implementation of an ICU recovery center. *J Crit Care* 2018, 46:141-148.

58. van Sleeuwen D, Zegers M, Ramjith J, Cruijsberg JK, Simons KS, van Bommel D, Burgers-Bonthuis D, Koeter J, Bisschops LLA, Janssen I *et al*: Prediction of Long-Term Physical, Mental, and Cognitive Problems Following Critical Illness: Development and External Validation of the PROSPECT Prediction Model. *Crit Care Med* 2024, 52(2):200-209.

59. Vialatte de Pémille C, Ray A, Michel A, Stefano F, Yim T, Bruel C, Zuber M: Prevalence and prospective evaluation of cognitive dysfunctions after SARS due to SARS-CoV-2 virus. The COgnitiVID study. *Rev Neurol (Paris)* 2022, 178(8):802-807.

60. Vincent A, Beck K, Thommen E, Widmer M, Becker C, Loretz N, Gross S, Mueller J, Amacher SA, Bohren C *et al*: Post-intensive care syndrome in out-of-hospital cardiac arrest patients: A prospective observational cohort study. *PLoS One* 2022, 17(10):e0276011.

61. Wilcox ME, McAndrews MP, Van J, Jackson JC, Pinto R, Black SE, Lim AS, Friedrich JO, Rubenfeld GD: Sleep Fragmentation and Cognitive Trajectories After Critical Illness. *Chest* 2021, 159(1):366-381.

62. Wood MD, Maslove DM, Muscedere J, Scott SH, Boyd JG: Robotic technology provides objective and quantifiable metrics of neurocognitive functioning in survivors of critical illness:A feasibility study. *J Crit Care* 2018, 48:228-236.

63. Yao L, Li Y, Yin R, Yang L, Ding N, Li B, Shen X, Zhang Z: Incidence and influencing factors of post-intensive care cognitive impairment. *Intensive Crit Care Nurs* 2021, 67:103106.

64. Chung CR, Yoo HJ, Park J, Ryu S: Cognitive Impairment and Psychological Distress at Discharge from Intensive Care Unit. *Psychiatry Investig* 2017, 14(3):376-379.

65. Elias MN, Ahrens EA, Tsai CS, Liang Z, Munro CL: Inactivity May Identify Older Intensive Care Unit Survivors at Risk for Post-Intensive Care Syndrome. *Am J Crit Care* 2024, 33(2):95-104.

66. Guerra C, Linde-Zwirble WT, Wunsch H: Risk factors for dementia after critical illness in elderly Medicare beneficiaries. *Crit Care* 2012, 16(6):R233.

67. Ko RE, Kang D, Park H, Cho J, Suh GY, Chung CR: Association between the presence of delirium during intensive care unit admission and cognitive impairment or psychiatric problems: the Korean ICU National Data Study. *J Intensive Care* 2022, 10(1):7.

68. Martillo MA, Dangayach NS, Tabacof L, Spielman LA, Dams-O'Connor K, Chan CC, Kohli-Seth R, Cortes M, Escalon MX: Postintensive Care Syndrome in Survivors of Critical Illness Related to Coronavirus Disease 2019: Cohort Study From a New York City Critical Care Recovery Clinic. *Crit Care Med* 2021, 49(9):1427-1438.

69. Mason CK, Adie SK, Shea MJ, Konerman MC, Thomas MP, McSparron JI, Iwashyna TJ, Prescott HC, Thompson AD: Post-intensive cardiac care outpatient long-term outreach clinic (PICCOLO clinic): Defining health care needs and outcomes among coronary care unit survivors. *Am Heart J Plus* 2024, 38.

70. Palakshappa JA, Callahan KE, Pajewski NM, Files DC, Willard JJ, Williamson JD: Detection of Cognitive Impairment after Critical Illness with the Medicare Annual Wellness Visit: A Cohort Study. *Ann Am Thorac Soc* 2021, 18(10):1702-1707.

71. Sturgill JL, Mayer KP, Kalema AG, Dave K, Mora S, Kalantar A, Carter DJ, Montgomery-Yates AA, Morris PE: Post-intensive care syndrome and pulmonary fibrosis in patients surviving ARDS-pneumonia of COVID-19 and non-COVID-19 etiologies. *Sci Rep* 2023, 13(1):6554.

72. Weidman K, LaFond E, Hoffman KL, Goyal P, Parkhurst CN, Derry-Vick H, Schenck E, Lief L: Post-Intensive Care Unit Syndrome in a Cohort of COVID-19 Survivors in New York City. *Ann Am Thorac Soc* 2022, 19(7):1158-1168.

73. Yanagi N, Kamiya K, Hamazaki N, Matsuzawa R, Nozaki K, Ichikawa T, Valley TS, Nakamura T, Yamashita M, Maekawa E *et al*: Post-intensive care syndrome as a predictor of mortality in patients with critical illness: A cohort study. *PLoS One* 2021, 16(3):e0244564.

74. Kim SJ, Park K, Kim K: Post-intensive care syndrome and health-related quality of life in long-term survivors of intensive care unit. *Aust Crit Care* 2023, 36(4):477-484.

75. Maley JH, Brewster I, Mayoral I, Siruckova R, Adams S, McGraw KA, Piech AA, Detsky M, Mikkelsen ME: Resilience in Survivors of Critical Illness in the Context of the Survivors' Experience and Recovery. *Ann Am Thorac Soc* 2016, 13(8):1351-1360.

76. Mateo Rodríguez E, Puchades Gimeno F, Ezzeddine Angulo A, Asensio Samper J, Saiz Ruiz C, López Alarcón MD: Postintensive care syndrome in COVID-19. Unicentric pilot study. Calm does not come after the storm. *Med Clin (Engl Ed)* 2022, 159(7):321-326.

77. Sylvestre A, Adda M, Maltese F, Lannelongue A, Daviet F, Parzy G, Coiffard B, Roch A, Loundou A, Baumstarck K *et al*: Long-term neurocognitive outcome is not worsened by of the use of venovenous ECMO in severe ARDS patients. *Ann Intensive Care* 2019, 9(1):82.

78. Austin TA, Thomas ML, Lu M, Hodges CB, Darowski ES, Bergmans R, Parr S, Pickell D, Catazaro M, Lantrip C *et al*: Meta-analysis of Cognitive Function Following Non-severe SARS-CoV-2 Infection. *Neuropsychol Rev* 2024.

79. Premraj L, Kannapadi NV, Briggs J, Seal SM, Battaglini D, Fanning J, Suen J, Robba C, Fraser J, Cho SM: Mid and long-term neurological and neuropsychiatric manifestations of post-COVID-19 syndrome: A meta-analysis. *J Neurol Sci* 2022, 434:120162.

80. Kim YW, Kim DY, Shin JC, Park CI, Lee JD: The changes of cortical metabolism associated with the clinical response to donepezil therapy in traumatic brain injury. *Clin Neuropharmacol* 2009, 32(2):63-68.

81. Sakusic A, Rabinstein AA: Cognitive outcomes after critical illness. *Curr Opin Crit Care* 2018, 24(5):410-414.

82. Abate SM, Ahmed Ali S, Mantfardo B, Basu B: Rate of Intensive Care Unit admission and outcomes among patients with coronavirus: A systematic review and Meta-analysis. *PLoS One* 2020, 15(7):e0235653.

83. Migliavaca CB, Stein C, Colpani V, Barker TH, Ziegelmann PK, Munn Z, Falavigna M: Meta-analysis of prevalence: I(2) statistic and how to deal with heterogeneity. *Res Synth Methods* 2022, 13(3):363-367.

**Table 1.** Summary of key characteristics of included studies (n = 58)

| Author, year | Location | Study design | Sample size | Male (%) | Mean  age | Illness severity  scoring systems | Cognitive function  measurements |
| --- | --- | --- | --- | --- | --- | --- | --- |
| Balasubramanian 2020 | India | Prospective | 136 | 66 | 56 (median) | APACHE: 26 (median)  SOFA: 12 (median) | RBANS Update questionnaire |
| Bladwin 2021 | US | Prospective | 185 | 48 | 74 | APACHE II: 29 | CAM-ICU and Mini-Cog test |
| Bark 2023 | Sweden | Prospective | 57 | 77 | 61 | SAPS III: 53 | MoCA |
| Bottom-Tanzer 2023 | US | Prospective | 126 | 64 | 53 | APACHE II: 21  APACHE III: 67 | Clinical examination by physicians |
| Brück 2019 | Sweden | Prospective | 100 | 76 | 54 (median) | APACHE II: 26 (median)  SAPS III: 48 (median) | IQCODE (screening), CANTAB, CFQ |
| Brück 2018 | Sweden | Prospective | 216 | 61 | 62 (median) | APACHE II: 8 for no sepsis; 13 for severe sepsis (median) | CFQ |
| Bulic 2020 | Australia | Prospective | 103 | 52 | 60 | APACHE II: 20 | MMSE |
| Carenzo 2024 | Italy | Prospective | 105 | 78 | 61 | APACHE II: 10 (median)  SOFA: 4 (median) | TMT-B and MoCA-Blind |
| Castro-Avila 2023 | Chile | Prospective | 252 | 65 | 57 (median) | NR | MoCA–Blind |
| Chung 2017 | South Korea | Retrospective | 30 | 70 | 61 (median) | SOFA: 7 (median) | Mini-Cog test |
| Costas-Carrera 2024 | Spain | Prospective | 102 | 70 | 66 | APACHE II: 13  SOFA: 6 (median) | MoCA, Digits forward and backward from the WAIS-III, Stroop Test, FCSRT, JLO, TMT, COWAT, BNT, CRQ |
| De Tanti 2023 | Italy | Prospective | 29 | 65 | 60 | NR | ACE-R |
| Dubin 2021 | US | Prospective | 165 | 48 | 65 (median) | NR | MoCA |
| Duggan 2017 | US | Prospective | 280 | 53 | 59 (median) | Modified SOFA: 4.8 (median) | BRIEF-A, subjective measure, and TMT Part B |
| Elias 2024 | US | Retrospective | 49 | 67 | 73 | APACHE III: 81.5 | The NIH Toolbox Cognition Battery, FICAT, DCCST |
| Fagerberg 2023 | Denmark | Prospective | 64 | 52 | 65 | NR | WAIS III, WMI, PSI, POI |
| Fernández-Gonzalo 2020 | Spain | Prospective | 156 | 62 | 63 | APACHE II: 17 (median)  SOFA: 7 (median) | NART Spanish version, Subtest of Digits, Spatial Span, Symbol Search from WAIS-III, RAVLT, BVRT, SCWT, TMT, FAS test |
| Ferrante 2018 | US | Prospective | 391 | 41 | 84 | NR | MMSE |
| Fjone 2024 | Norway | Prospective | 684 | 70 | 60 (median) | SAPS II: 31 (median) | The Mini MoCA |
| Geense 2021 | Netherlands | Prospective | 3320 | 66 | 63 | APACHE IV: 62 for medical; 58 for urgent surgical; 50 for elective surgical | 14-item CFQ |
| Godoy-González 2023 | Spain | Prospective | 80 | 69 | 60 (median) | APACHE II: 8 (median) | IQCODE (screening), Digit Span Forward and Backward from the WAIS-III, Spatial Score Forward and Backward from the WMS-III, RAVLT, SPART, SCWT, TMT Part A and B, CTT, FAS, NART, Vocabulary subtest of the WAIS-IV, PDQ, CRQ |
| Guerra 2012 | US | Retrospective | 25368 | 48 | 77 | NR | ICD 9^th^ edition |
| Habib 2014 | Pakistan | Prospective | 138 | 84 | 54 | NR | MMSE, McNair's and Kahn Auto-evaluation (Urdu translation) |
| Haddad 2020 | US | Prospective | 1040 | 60 | 62 (median) | APACHE II: 24 (median) | CDR (screening), IQCODE |
| Hatakeyama 2022 | Japan | Prospective | 334 | 80 | 68 (median) | SOFA: 5 (median) | IQCODE |
| Jackson 2011 | US | Prospective | 173 | 57 | 43 | NR | IQCODE |
| Jaquet 2022 | France | Prospective | 41 | 76 | 56 (median) | SAPS II: 30 (median) | MoCA |
| Jones 2006 | UK | Prospective | 30 | 57 | 54 (median) | APACHE II: 16 (median) | CANTAB |
| Kang 2024 | South Korea | Prospective | 475 | 59 | 61 | APACHE II: 12  SAPS III: 33 | MoCA-Blind |
| Karnatovskaia 2019 | US | Prospective | 300 | 57 | 53 | APACHE III: 32-49 (median)  SOFA: 4-8 (median) | MoCA-Blind |
| Kawakami 2021 | Japan | Prospective | 192 | 65 | 74 (median) | APACHE II: 23 (median)  SOFA: 8 (median) | SMQ |
| Kim 2023 | South Korea | Cross-sectional | 131 | 59 | 64 | APACHE II: 18 | The Korean-MoCA |
| Klinkhammer 2023 | Netherlands | Prospective | 205 | 70 | 63 | APACHE IV: 55  SOFA: 7 (median) | MoCA, TMT, Stroop, COWA, Category Fluency, Digit Span, Symbol Digit Substitution, RAVLT, JLO, BNT |
| Ko 2022 | South Korea | Retrospective | 306011 | 65 | 58 | NR | ICD |
| Kosilek 2021 | Germany | Prospective | 289 | 67 | 64 (median) | NR | TICS-M |
| Maley 2016 | US | Cross-sectional | 45 | 42 | 59 | NR | HUI-3 |
| Mart 2024 | US | Prospective | 501 | 59 | 50+ (median)^a^ | SOFA: 5 for lower SpO_2_; 4 for intermediate and higher SpO_2_ (median) | Telephone MoCA |
| Martillo 2021 | US | Retrospective | 49 | 73 | 54 | SOFA: 4.5 (median) | Telephone MoCA |
| Martínez 2023 | Argentina | Prospective | 40 | 75 | 69 (median) | APACHE II: 19 (median)  SOFA: 8 (median) | MoCA |
| Mason 2024 | US | Retrospective | 134 | 76 | 65 | NR | MoCA |
| Mateo 2022 | Spain | Cross-sectional | 29 | 55 | 63 | SOFA: 3 (median) | MoCA |
| Mimenza 2024 | Mexico | Prospective | 107 | 42 | 70 | NR | MoCA |
| Mitchell 2018 | Australia | Prospective | 148 | 69 | 57 (median) | APACHE II: 18  APACHE III: 57 | RBANS, TMT Part A and B, MMSE |
| Nordness 2021 | US | Prospective | 590 | 59 | 61 (median) | SOFA: 6 (median) | RBANS, TMT Part B |
| Palakshappa 2021 | US | Retrospective | 696 | 49 | 75 | NR | ICD 10^th^ edition |
| Pereira 2018 | Portugal | Prospective | 267 | 56 | 62 | SAPS II: 44  SOFA: 3 (median) | DRS-2 |
| Proffitt 2023 | US | Prospective | 50 | 52 | 59 | NR | MoCA |
| Sevin 2018 | US | Prospective | 162 | 48 | 54 | SOFA: 10 (median) | MoCA, TMT Part A and B |
| Sturgill 2023 | US | Retrospective | 94 | 51 | 53 | SOFA: 11 | MoCA |
| Sylvestre 2019 | France | Cross-sectional | 40 | 58 | 41+ (median)^b^ | SAPS II: 43 for non-ECMO; 42 for ECMO  SOFA: 8 (median) | WAIS-IV |
| van Sleeuwen 2024 | Netherlands | Prospective | 2476 | 62 | 61 | APACHE: 56 | abbreviated CFQ-14 |
| Vialatte de Pémille 2022 | France | Prospective | 13 | 39 | 62 (median) | NR | MMSE, FAB; 40 words oral naming test, Dubois five words test, forwards or backwards digit spans; similarities test of the WAIS-IV, Brixton test, SCWT-Victoria version; categorical and lexical verbal fluencies during two minutes |
| Vincent 2022 | Switzerland | Prospective | 156 | 83 | 63 (median) | APACHE II: 25 (median)  SAPS II: 58 (median) | CPC, mRS |
| Weidman 2022 | US | Retrospective | 87 | 74 | 62 (median) | SOFA: 12 (median) | MoCA or MoCA blind (for video visits) |
| Wilcox 2021 | Canada | Prospective | 150 | 54 | 57 (median) | APACHE III: 57 for surviving patients; 75 for patients who died or withdrew (median) | RBANS, TMT Part A and B, IQCODE-SF |
| Wood 2018 | Canada | Prospective | 70 | 69 | 67 (median) | APACHE: 19 (median) | RBANS |
| Yanagi 2021 | Japan | Retrospective | 248 | 70 | 69 (median) | APACHE II: 16 (median) | Mini-Cog test |
| Yao 2021 | China | Prospective | 431 | 59 | 55 | APACHE II >15: n = 158 (39%)  SOFA >4: n = 31 (8%) | MoCA |

*APACHE* Acute Physiology and Chronic Health Evaluation, *SAPS* Simplified Acute Physiology Score, *SOFA* Sequential Organ Failure Assessment, *RBANS* Repeatable Battery for the Assessment of Neuropsychological Status, *CAM-ICU* Confusion Assessment Method for the intensive care unit, *MoCA* Montreal Cognitive Assessment, *IQCODE* The Informant Questionnaire on Cognitive Decline in the Elderly, *CANTAB* Cambridge Neuropsychological Test Automated Battery, *CFQ* Cognitive Failures Questionnaire, *MMSE* Mini-Mental State Examination, *TMT* Trail Making Test, *WAIS* Wechsler Adult Intelligence Scale, *FCSRT* Free Cued Selective Reminding Test, *JLO* Judgement of Line Orientation, *COWAT* Controlled Oral Word Association Test, *BNT* Boston naming test, *CRQ* Cognitive Reserve Questionnaire, *ACE-R* Addenbrooke’s Cognitive Examination Revised, *BRIEF-A* Behavior Rating Inventory of Executive Function-Adult, *NIH* National Institutes of Health, *FICAT* The Flanker Inhibitory Control and Attention Test, *DCCST* Dimensional Change Card Sort Test, *WMI* Working Memory Index, *PSI* Processing Speed Index, *POI* Perceptual Organization Index, *NART* National Adult Reading Test, *RAVLT* Rey Auditory Verbal Learning Test, *BVRT* Benton Visual Retention Test, *SCWT* Stroop Color and Word Test, *FAS* verbal fluency test, *WMS* Wechsler Memory Scale, *SPART* Spatial Recall Test, *CTT* Color Trails Test, *NART* The National Adult Reading Test, *PDQ* Perceived Deficits Questionnaire, *CRQ* Cognitive Reserve Questionnaire, *ICD* International Classification of Diseases, *CDR* Clinical Dementia Rating, *SMQ* Short-Memory Questionnaire, *TICS-M* Telephone Interview of Cognitive Status, *HUI-3* Health Utilities Index-3, *DRS* Dementia Rating Scale, *FAB* Frontal Assessment Battery, *CPC* Cerebral Performance Category, *mRS* modified Rankin Scale, *NR* not reported, *ECMO* Extracorporeal membrane oxygenation.

^a^ Median age in the lower SpO_2_ group: 55 (n = 142), intermediate SpO2 group: 58 (n = 186), and higher SpO_2_ group: 51 (n = 173).

^b^ Median age in the non-ECMO group: 51 (n = 18), and ECMO group: 41 (n = 22).

**Identification of studies via other methods**

**Identification of studies via databases and registers**

Records identified from:

Citation searching (n = 38)

Records removed *before screening*:

Duplicate records removed

(n = 1783)

Records identified from:

Databases (n = 9193)

CINAHL Plus = 74

Cochrane Library = 356

EMBASE = 1547

PsycINFO = 879

PubMed = 3741

Web of Science = 2596

**Identification**

Records excluded

(n = 7254)

Main reasons for exclusion at screening:

- Not adult population (PICU/NICU/Pediatric patients)
- Language: Non-English

Records screened

(n = 7410)

Reports sought for retrieval

(n = 156)

Reports assessed for eligibility

(n = 38)

**Screening**

Reports assessed for eligibility

(n = 118)

Reports excluded:

Not post-ICU setting (n = 37)

No cognitive impairment data (n = 16)

Same population (n = 3)

Non-English (n = 4)

Studies included in review

(n = 58)

Reports of included studies

(n = 58)

**Included**

**Figure 1.** PRISMA 2020 Flow Diagram

**
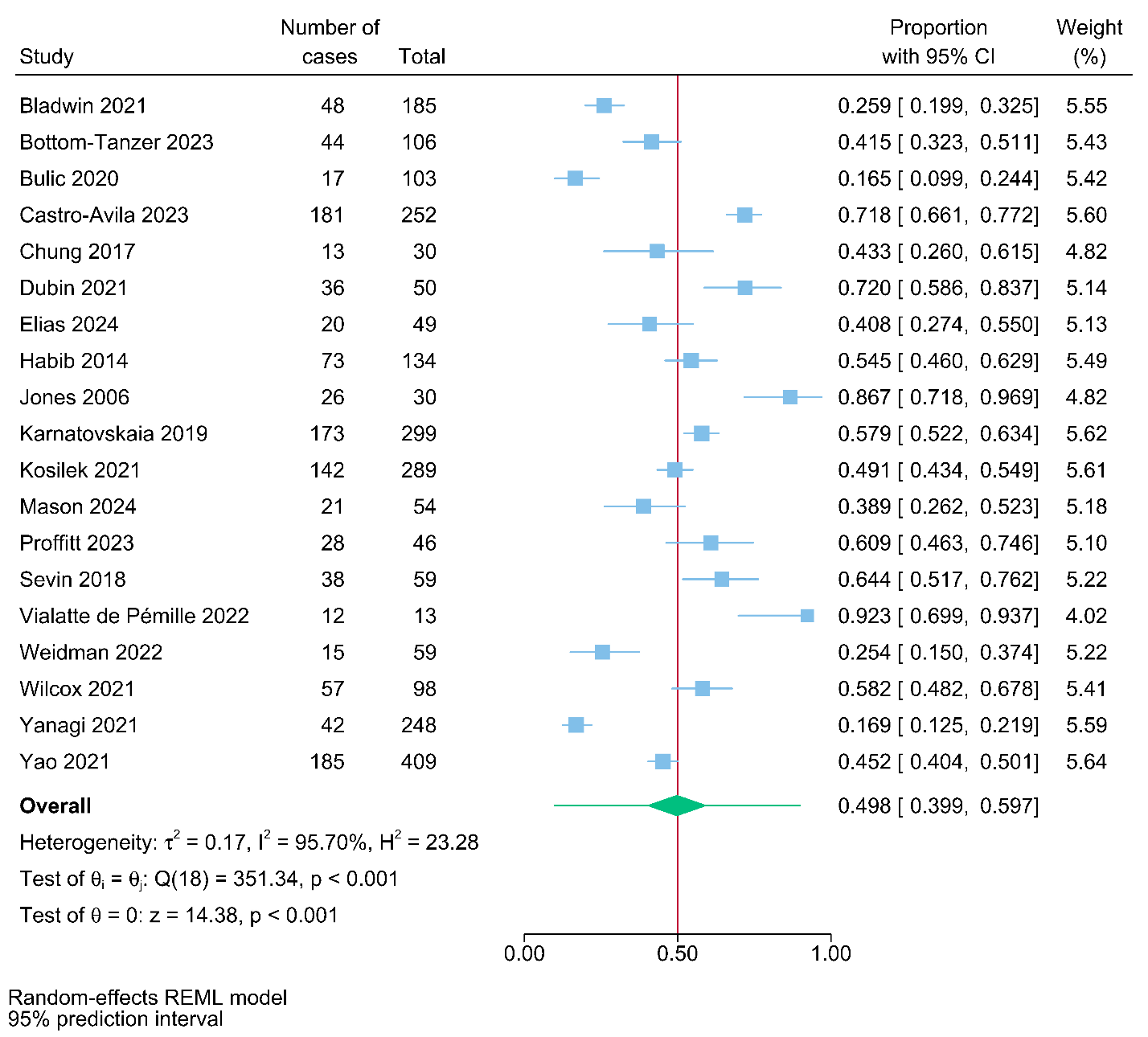
**

**Figure 2.** Pooled Proportions of Post-Intensive Care Cognitive Impairment at Follow-up within a Month


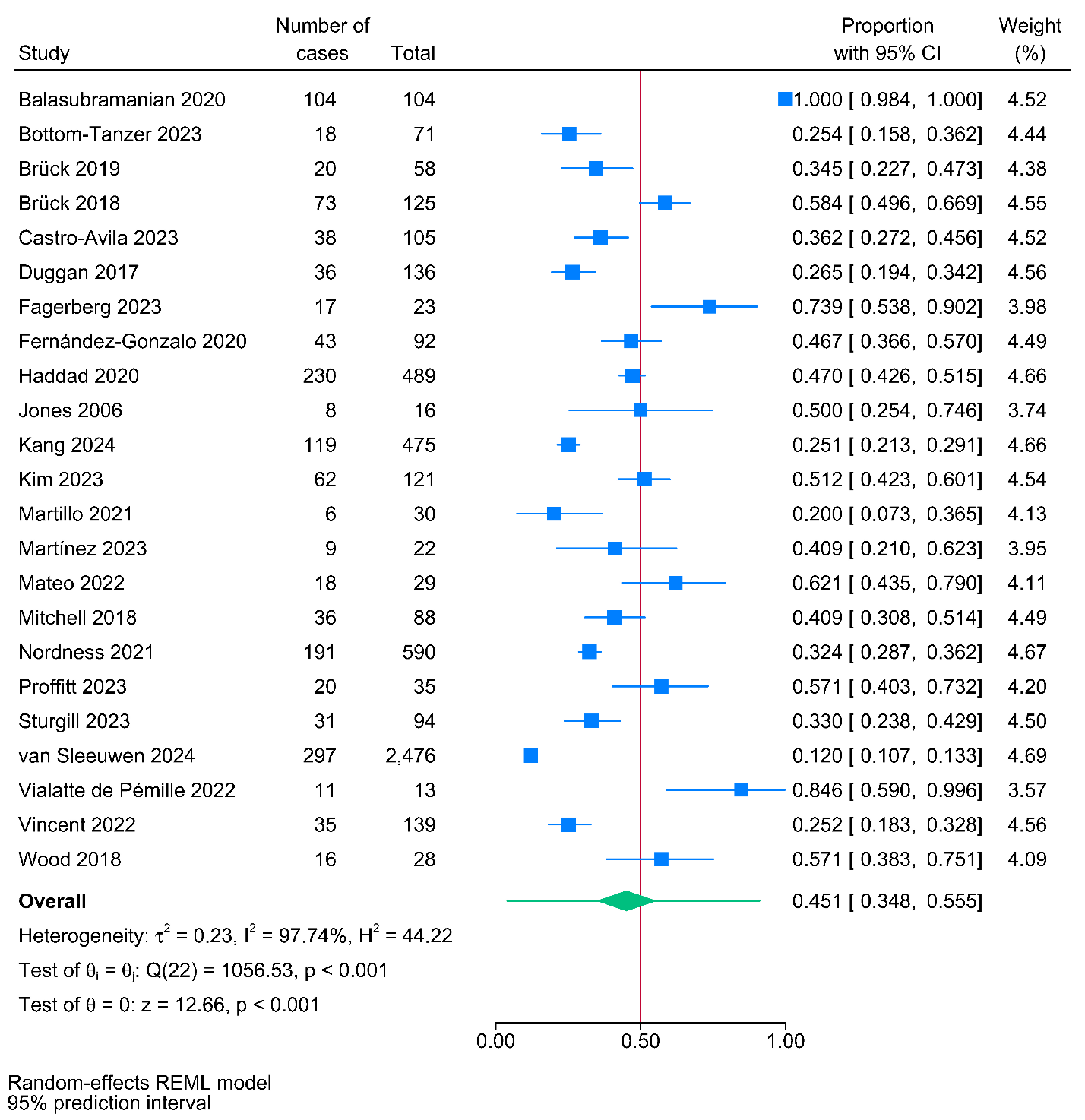


**Figure 3.** Pooled Proportions of Post-Intensive Care Cognitive Impairment at 1 to 3 Month(s) Follow-up


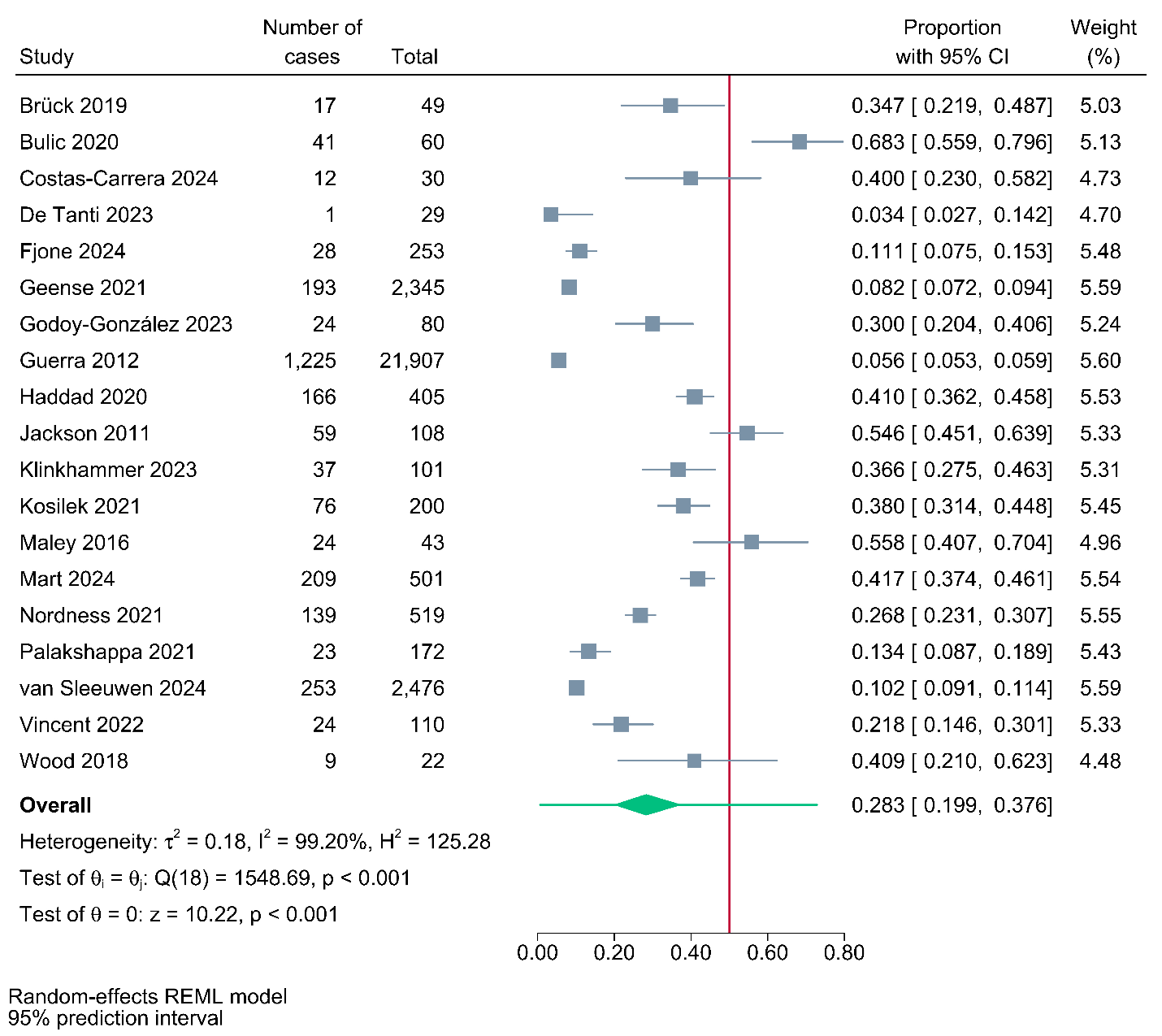


**Figure 4.** Pooled Proportions of Post-Intensive Care Cognitive Impairment at 7 to 12 Months Follow-up

**A**


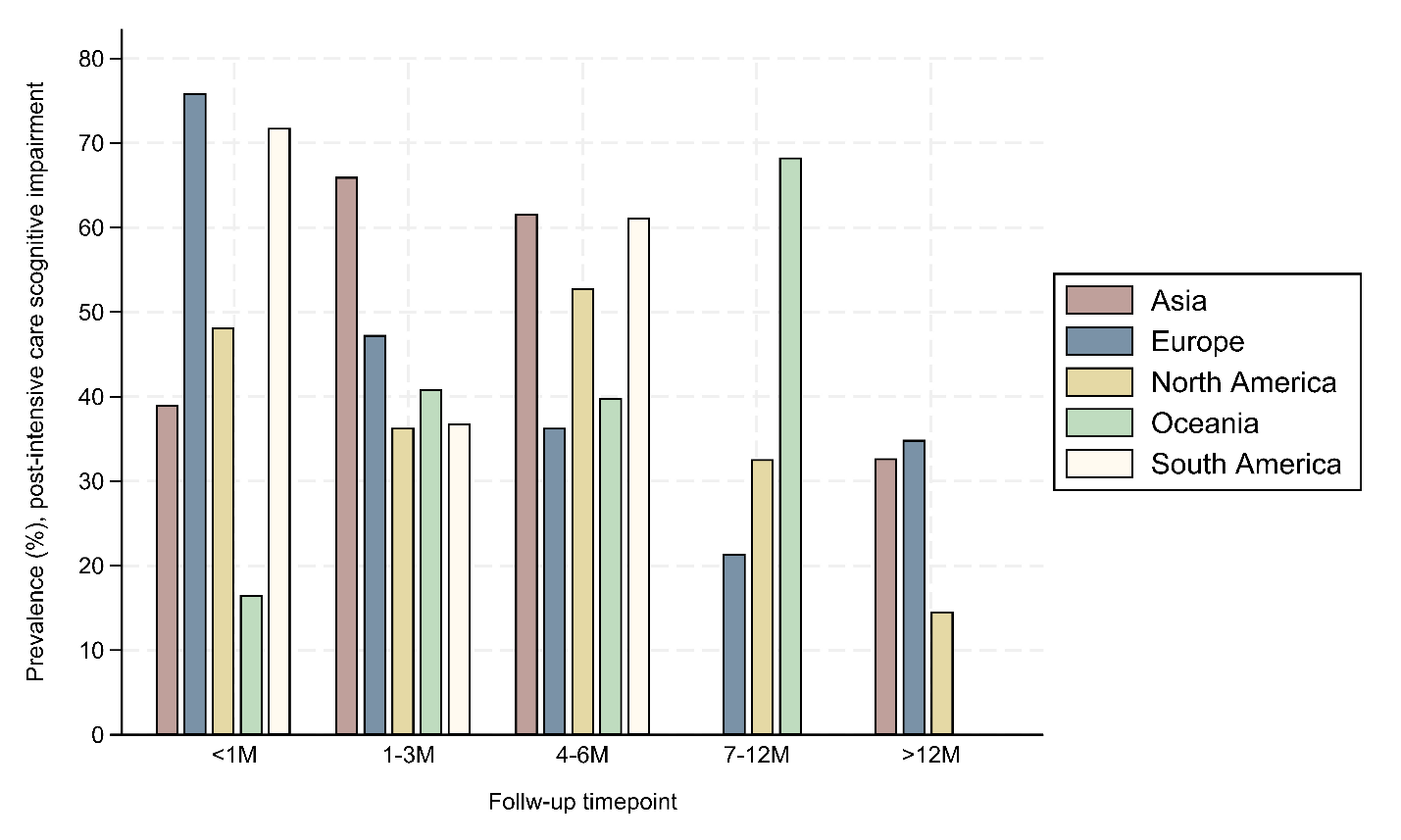


**B**


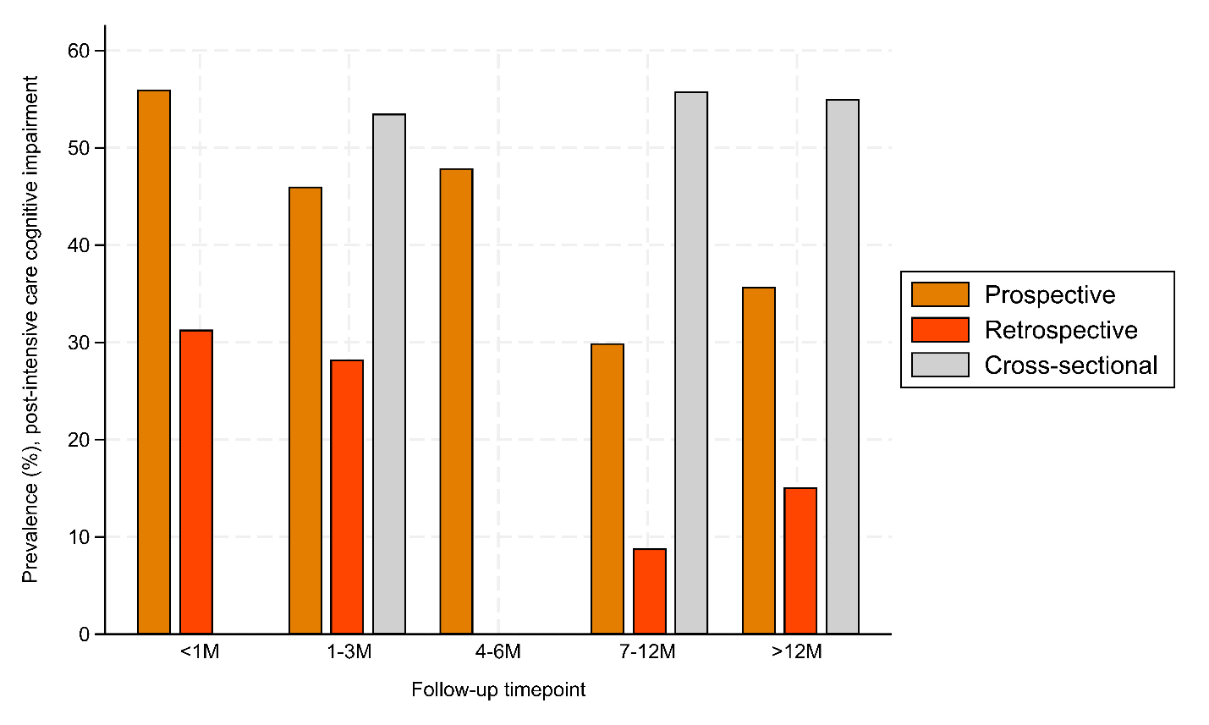


**Figure 5.** Pooled Prevalence Rates of Post-Intensive Care Cognitive Impairment in Different Continents (Figure 5A) and Study Designs (Figure 5B) Subgroups Across all Follow-up Timepoints

**Supplementary Materials**

**Table of contents**

**eTable 1.** PRISMA 2020 Checklist

**eTable 2.** Search Strategy

**eTable 3.** Risk of Bias Assessment According to Newcastle-Ottawa Scale

**eFigure 1.** Pooled Proportions of Post-Intensive Care Cognitive Impairment at 4 to 6 Months Follow-up

**eFigure 2.** Pooled Proportions of Post-Intensive Care Cognitive Impairment at over 12 Months Follow-up

**eFigure 3.** Subgroup Analyses on Post-Intensive Care Cognitive Impairment Prevalence at Follow-Up Within a Month

**eFigure 4.** Subgroup Analyses on Post-Intensive Care Cognitive Impairment Prevalence at 1 to 3 Month(s) Follow-Up

**eFigure 5.** Subgroup Analyses on Post-Intensive Care Cognitive Impairment Prevalence at 4 to 6 Months Follow-Up

**eFigure 6.** Subgroup Analyses on Post-Intensive Care Cognitive Impairment Prevalence at 7 to 12 Months Follow-Up

**eFigure 7.** Subgroup Analyses on Post-Intensive Care Cognitive Impairment Prevalence at Follow-Up Over 12 Month

**eFigure 8.** Leave-One-Out Meta-Analysis on Post-Intensive Care Cognitive Impairment Prevalence at Follow-Up Within a Month

**eFigure 9.** Leave-One-Out Meta-Analysis on Post-Intensive Care Cognitive Impairment Prevalence at 1 to 3 Month(s) Follow-Up

**eFigure 10.** Leave-One-Out Meta-Analysis on Post-Intensive Care Cognitive Impairment Prevalence at 4 to 6 Months Follow-Up

**eFigure 11.** Leave-One-Out Meta-Analysis on Post-Intensive Care Cognitive Impairment Prevalence at 7 to 12 Months Follow-Up

**eFigure 12.** Leave-One-Out Meta-Analysis on Post-Intensive Care Cognitive Impairment Prevalence at Follow-Up Over 12 Months


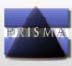


**eTable 1.** PRISMA 2020 Checklist

| **Section and Topic** | **Item #** | **Checklist item** | **Location where item is reported** |
| --- | --- | --- | --- |
| **TITLE** | | |  |
| Title | 1 | Identify the report as a systematic review. |  |
| **ABSTRACT** | | |  |
| Abstract | 2 | See the PRISMA 2020 for Abstracts checklist. |  |
| **INTRODUCTION** | | |  |
| Rationale | 3 | Describe the rationale for the review in the context of existing knowledge. |  |
| Objectives | 4 | Provide an explicit statement of the objective(s) or question(s) the review addresses. |  |
| **METHODS** | | |  |
| Eligibility criteria | 5 | Specify the inclusion and exclusion criteria for the review and how studies were grouped for the syntheses. |  |
| Information sources | 6 | Specify all databases, registers, websites, organisations, reference lists and other sources searched or consulted to identify studies. Specify the date when each source was last searched or consulted. |  |
| Search strategy | 7 | Present the full search strategies for all databases, registers and websites, including any filters and limits used. |  |
| Selection process | 8 | Specify the methods used to decide whether a study met the inclusion criteria of the review, including how many reviewers screened each record and each report retrieved, whether they worked independently, and if applicable, details of automation tools used in the process. |  |
| Data collection process | 9 | Specify the methods used to collect data from reports, including how many reviewers collected data from each report, whether they worked independently, any processes for obtaining or confirming data from study investigators, and if applicable, details of automation tools used in the process. |  |
| Data items | 10a | List and define all outcomes for which data were sought. Specify whether all results that were compatible with each outcome domain in each study were sought (e.g. for all measures, time points, analyses), and if not, the methods used to decide which results to collect. |  |
|  | 10b | List and define all other variables for which data were sought (e.g. participant and intervention characteristics, funding sources). Describe any assumptions made about any missing or unclear information. |  |
| Study risk of bias assessment | 11 | Specify the methods used to assess risk of bias in the included studies, including details of the tool(s) used, how many reviewers assessed each study and whether they worked independently, and if applicable, details of automation tools used in the process. |  |
| Effect measures | 12 | Specify for each outcome the effect measure(s) (e.g. risk ratio, mean difference) used in the synthesis or presentation of results. |  |
| Synthesis methods | 13a | Describe the processes used to decide which studies were eligible for each synthesis (e.g. tabulating the study intervention characteristics and comparing against the planned groups for each synthesis (item #5)). |  |
|  | 13b | Describe any methods required to prepare the data for presentation or synthesis, such as handling of missing summary statistics, or data conversions. |  |
|  | 13c | Describe any methods used to tabulate or visually display results of individual studies and syntheses. |  |
|  | 13d | Describe any methods used to synthesize results and provide a rationale for the choice(s). If meta-analysis was performed, describe the model(s), method(s) to identify the presence and extent of statistical heterogeneity, and software package(s) used. |  |
|  | 13e | Describe any methods used to explore possible causes of heterogeneity among study results (e.g. subgroup analysis, meta-regression). |  |
|  | 13f | Describe any sensitivity analyses conducted to assess robustness of the synthesized results. |  |
| Reporting bias assessment | 14 | Describe any methods used to assess risk of bias due to missing results in a synthesis (arising from reporting biases). |  |
| Certainty assessment | 15 | Describe any methods used to assess certainty (or confidence) in the body of evidence for an outcome. |  |
| **RESULTS** | | |  |
| Study selection | 16a | Describe the results of the search and selection process, from the number of records identified in the search to the number of studies included in the review, ideally using a flow diagram. |  |
|  | 16b | Cite studies that might appear to meet the inclusion criteria, but which were excluded, and explain why they were excluded. |  |
| Study characteristics | 17 | Cite each included study and present its characteristics. |  |
| Risk of bias in studies | 18 | Present assessments of risk of bias for each included study. |  |
| Results of individual studies | 19 | For all outcomes, present, for each study: (a) summary statistics for each group (where appropriate) and (b) an effect estimate and its precision (e.g. confidence/credible interval), ideally using structured tables or plots. |  |
| Results of syntheses | 20a | For each synthesis, briefly summarise the characteristics and risk of bias among contributing studies. |  |
|  | 20b | Present results of all statistical syntheses conducted. If meta-analysis was done, present for each the summary estimate and its precision (e.g. confidence/credible interval) and measures of statistical heterogeneity. If comparing groups, describe the direction of the effect. |  |
|  | 20c | Present results of all investigations of possible causes of heterogeneity among study results. |  |
|  | 20d | Present results of all sensitivity analyses conducted to assess the robustness of the synthesized results. |  |
| Reporting biases | 21 | Present assessments of risk of bias due to missing results (arising from reporting biases) for each synthesis assessed. |  |
| Certainty of evidence | 22 | Present assessments of certainty (or confidence) in the body of evidence for each outcome assessed. |  |
| **DISCUSSION** | | |  |
| Discussion | 23a | Provide a general interpretation of the results in the context of other evidence. |  |
|  | 23b | Discuss any limitations of the evidence included in the review. |  |
|  | 23c | Discuss any limitations of the review processes used. |  |
|  | 23d | Discuss implications of the results for practice, policy, and future research. |  |
| **OTHER INFORMATION** | | |  |
| Registration and protocol | 24a | Provide registration information for the review, including register name and registration number, or state that the review was not registered. |  |
|  | 24b | Indicate where the review protocol can be accessed, or state that a protocol was not prepared. |  |
|  | 24c | Describe and explain any amendments to information provided at registration or in the protocol. |  |
| Support | 25 | Describe sources of financial or non-financial support for the review, and the role of the funders or sponsors in the review. |  |
| Competing interests | 26 | Declare any competing interests of review authors. |  |
| Availability of data, code and other materials | 27 | Report which of the following are publicly available and where they can be found: template data collection forms; data extracted from included studies; data used for all analyses; analytic code; any other materials used in the review. |  |

*From:*  Page MJ, McKenzie JE, Bossuyt PM, Boutron I, Hoffmann TC, Mulrow CD, et al. The PRISMA 2020 statement: an updated guideline for reporting systematic reviews. BMJ 2021;372:n71. doi: 10.1136/bmj.n71

**eTable 2.** Search Strategy

| **Cochrane Library** | | |
| --- | --- | --- |
| ID | Search queries | Results |
| #1 | MeSH descriptor: [Cognitive Dysfunction] explode all trees | 4,093 |
| #2 | MeSH descriptor: [Cognition] explode all trees | 16,398 |
| #3 | MeSH descriptor: [Cognition Disorders] explode all trees | 8,545 |
| #4 | (cogniti* or cognitive impairment or "post-intensive care" or "post-intensive care cognitive" or "post-intensive cognitive dysfunction"):ti,ab,kw | 2,748 |
| #5 | MeSH descriptor: [Intensive Care Units] explode all trees | 6,123 |
| #6 | MeSH descriptor: [Critical Illness] explode all trees | 432 |
| #7 | (intensive care or critical care "discharge" or "post ICU" or "ICU discharge"):ti,ab,kw | 5,621 |
| #8 | (#1 or #2 or #3 or #4) AND (#5 or #6 or #7) | 356 |
| Trials |  | 312 |
| **PubMed** | | |
| ID | Search queries | Results |
| #1 | (Cognitive Dysfunction[MeSH Terms]) OR (Cognition[MeSH Terms]) OR (Cognition Disorders[MeSH Terms]) | [15,099](https://pubmed.ncbi.nlm.nih.gov/?term=%28%28%28%28Dementia%5BMeSH+Terms%5D%29+OR+%28%22Alzheimer+Disease%22%5BMeSH+Terms%5D%29%29+OR+%28%22Cognitive+Dysfunction%22%5BMeSH+Terms%5D%29%29+OR+%28%22Neurocognitive+Disorders%22%5BMeSH+Terms%5D%29%29+OR+%28%22Cognition+Disorders%22%5BMeSH+Terms%5D%29&sort=) |
| #2 | "cogniti*"[Title/Abstract] OR "cognitive impairment"[Title/Abstract] OR "post-intensive care cognitive impairment"[Title/Abstract] | [30,](https://pubmed.ncbi.nlm.nih.gov/?term=deliri%2A%5BTitle%2FAbstract%5D+OR+hallucinat%2A%5BTitle%2FAbstract%5D+OR+%22acute+confusion%22%5BTitle%2FAbstract%5D+OR+%22acute+organic+psychosyndrome%22%5BTitle%2FAbstract%5D+OR+%22acute+brain+syndrome%22%5BTitle%2FAbstract%5D+OR+%22acute+psycho-organic+syndrome%22%5BTitle%2FAbstract%5D+OR+%22exogenous+psychosis%22%5BTitle%2FAbstract%5D+OR+%22toxic+psychosis%22%5BTitle%2FAbstract%5D+OR+%22toxic+confusion%22%5BTitle%2FAbstract%5D+OR+obnubilate%2A%5BTitle%2FAbstract%5D&sort=)749 |
| #3 | "Intensive Care Units"[MeSH Terms] OR "Critical Illness"[MeSH Terms] | 5,355 |
| #4 | "intensive care"[Title/Abstract] OR "post intensive care"[Title/Abstract] OR "post ICU"[Title/Abstract] OR "discharge"[Title/Abstract] OR "ICU discharge"[Title/Abstract] | [20,](https://pubmed.ncbi.nlm.nih.gov/?term=stroke%5BTitle%2FAbstract%5D+OR+%22post+stroke%22%5BTitle%2FAbstract%5D+OR+%22cerebrovascular+accident%22%5BTitle%2FAbstract%5D+OR+%22cerebrovascular+event%22%5BTitle%2FAbstract%5D+OR+%22apoplexy%22%5BTitle%2FAbstract%5D+OR+%22brain+vascular+accident%22%5BTitle%2FAbstract%5D+OR+%22brain+attack%22%5BTitle%2FAbstract%5D+OR+%22transient+ischaemic+attack%22%5BTitle%2FAbstract%5D+OR+%22transient+ischemic+attack%22%5BTitle%2FAbstract%5D&sort=)031 |
| #5 | (#1 or #2) AND (#3 or #4) | 3,741 |
| **EMBASE** | | |
| ID | Search queries | Results |
| #1 | Cognitive impairment/ | 148,925 |
| #2 | "Cognitive Defect"/ | 234,070 |
| #3 | Post-Intensive Care Cognitive Impairment/ | 2 |
| #4 | (cogniti* or cognitive impairment or "post-intensive care" or "post-intensive care cognitive" or "post-intensive cognitive dysfunction").ti,ab,kw. | 354,747 |
| #5 | "Intensive Care Unit" | 252,656 |
| #6 | (intensive care or critical care "discharge" or "post ICU" or "ICU discharge"):ti,ab,kw | 368 |
| #7 | 1 or 2 or 3 or 4 | 512,153 |
| #8 | 5 or 6 | 273,422 |
| #9 | 7 and 8 | 1,547 |
| **PsycINFO** | | |
|  | (SU(Cogniti* or "Cognitive Impairment" or "Cognitive Dysfunction" or " Cognition Disorders) OR (Cognitive Defect or post-intensive care cognitive impairment or "post-ICU cognitive impairment")) AND (SU(Intensive Care Units OR "Critical Care" OR "ICU Discharge" OR "Post ICU" OR "ICU Dscharge") | 879 |

*(continued)*

| **CINAHL Plus** | | |
| --- | --- | --- |
|  | (SU(Cogniti* or "Cognitive Impairment" or "Cognitive Dysfunction" or " Cognition Disorders) OR (Cognitive Defect or post-intensive care cognitive impairment or "post-ICU cognitive impairment")) AND (SU(Intensive Care Units OR "Critical Care" OR "ICU Discharge" OR "Post ICU" OR "ICU Dscharge") | 74 |
| **Web of Science** | | |
|  | TS=(Cogniti* or "Cognitive Impairment" or "Cognitive Dysfunction" or " Cognition Disorders) AND TS=(Cognitive Defect or post-intensive care cognitive impairment or "post-ICU cognitive impairment")) AND TS=(Intensive Care Units OR "Critical Care" OR "ICU Discharge" OR "Post ICU" OR "ICU Dscharge") | 2596 |

**eTable 3.** Risk of Bias Assessment According to Newcastle-Ottawa Scale

| First author, year | Selection | Comparability | Outcome | Total Score | Decision |
| --- | --- | --- | --- | --- | --- |
| Balasubramanian 2020^a^ | ★★★☆ | ★☆ | ★★★ | 7/9 | Included |
| Bladwin 2021^a^ | ★★★★ | ★★ | ★★★ | 9/9 | Included |
| Bark 2023^a^ | ★★★☆ | ★☆ | ★★★ | 7/9 | Included |
| Bottom-Tanzer 2023^a^ | ★★★☆ | ★★ | ★★★ | 8/9 | Included |
| Brück 2019^a^ | ★★★★ | ★★ | ★★★ | 9/9 | Included |
| Brück 2018^a^ | ★★★★ | ★★ | ★★★ | 9/9 | Included |
| Bulic 2020^a^ | ★★★★ | ★★ | ★★★ | 9/9 | Included |
| Carenzo 2024^a^ | ★★★★ | ★★ | ★★★ | 9/9 | Included |
| Castro-Avila 2023^a^ | ★★★☆ | ★★ | ★★★ | 8/9 | Included |
| Chung 2017^a^ | ★★★★ | ★★ | ★★★ | 9/9 | Included |
| Costas-Carrera 2024^a^ | ★★★★ | ★★ | ★★★ | 9/9 | Included |
| De Tanti 2023^a^ | ★★★☆ | ★☆ | ★★★ | 7/9 | Included |
| Dubin 202 1^a^ | ★★★☆ | ★★ | ★★★ | 8/9 | Included |
| Duggan 2017^a^ | ★★★★ | ★★ | ★★★ | 9/9 | Included |
| Elias 2024^a^ | ★★★★ | ★★ | ★★★ | 9/9 | Included |
| Fagerberg 2023^a^ | ★★★★ | ★★ | ★★★ | 9/9 | Included |
| Fernández-Gonzalo 2020^a^ | ★★★★ | ★★ | ★★★ | 9/9 | Included |
| Ferrante 2018^a^ | ★★★★ | ★★ | ★★★ | 9/9 | Included |
| Fjone 2024^a^ | ★★★☆ | ★★ | ★★★ | 8/9 | Included |
| Geense 2021^a^ | ★★★★ | ★★ | ★★★ | 9/9 | Included |
| Godoy-González 2023^a^ | ★★★★ | ★★ | ★★★ | 9/9 | Included |
| Guerra 2012^a^ | ★★★★ | ★★ | ★★★ | 9/9 | Included |
| Habib 2014^a^ | ★★★☆ | ★☆ | ★★★ | 7/9 | Included |
| Haddad 2020^a^ | ★★★★ | ★★ | ★★★ | 9/9 | Included |
| Hatakeyama 2022^a^ | ★★★☆ | ★★ | ★★★ | 8/9 | Included |
| Jackson 2011^a^ | ★★★★ | ★★ | ★★★ | 9/9 | Included |
| Jaquet 2022^a^ | ★★★★ | ★★ | ★★★ | 9/9 | Included |
| Jones 2006^a^ | ★★★★ | ★★ | ★★★ | 9/9 | Included |
| Kang 2024^a^ | ★★★★ | ★★ | ★★★ | 9/9 | Included |
| Karnatovskaia 2019^a^ | ★★★★ | ★★ | ★★★ | 9/9 | Included |
| Kawakami 2021^a^ | ★★★★ | ★★ | ★★★ | 9/9 | Included |
| Kim 2023^b^ | ★★☆☆ | ★★ | ★★★ | 7/10 | Included |
| Klinkhammer 2023^a^ | ★★★★ | ★★ | ★★★ | 9/9 | Included |
| Ko 2022^a^ | ★★★★ | ★★ | ★★★ | 9/9 | Included |
| Kosilek 2021^a^ | ★★★★ | ★★ | ★★★ | 9/9 | Included |
| Maley 2016^b^ | ★★☆☆ | ★★ | ★★★ | 7/10 | Included |
| Mart 2024^a^ | ★★★★ | ★★ | ★★★ | 9/9 | Included |
| Martillo 2021^a^ | ★★★★ | ★★ | ★★★ | 9/9 | Included |
| Martínez 2023^a^ | ★★★☆ | ★★ | ★★★ | 8/9 | Included |
| Mason 2024^a^ | ★★★★ | ★★ | ★★★ | 9/9 | Included |
| Mateo 2022^b^ | ★★☆☆ | ★★ | ★★★ | 7/10 | Included |
| Mimenza 2024^a^ | ★★★☆ | ★☆ | ★★★ | 7/9 | Included |
| Mitchell 2018^a^ | ★★★★ | ★★ | ★★★ | 9/9 | Included |
| Nordness 2021^a^ | ★★★★ | ★★ | ★★★ | 9/9 | Included |
| Palakshappa 2021^a^ | ★★★★ | ★★ | ★★★ | 9/9 | Included |
| Pereira 2018^a^ | ★★★☆ | ★★ | ★★★ | 8/9 | Included |
| Proffitt 2023^a^ | ★★★★ | ★★ | ★★★ | 9/9 | Included |
| Sevin 2018^a^ | ★★★★ | ★★ | ★★★ | 9/9 | Included |
| Sturgill 2023^a^ | ★★★☆ | ★★ | ★★★ | 8/9 | Included |
| Sylvestre 2019^b^ | ★★☆☆ | ★★ | ★★★ | 7/10 | Included |
| van Sleeuwen 2024^a^ | ★★★★ | ★★ | ★★★ | 9/9 | Included |
| Vialatte de Pémille 2022^a^ | ★★★☆ | ★★ | ★★★ | 8/9 | Included |
| Vincent 2022^a^ | ★★★★ | ★★ | ★★★ | 9/9 | Included |
| Weidman 2022^a^ | ★★★★ | ★★ | ★★★ | 9/9 | Included |
| Wilcox 2021^a^ | ★★★★ | ★★ | ★★★ | 9/9 | Included |
| Wood 2018^a^ | ★★★★ | ★★ | ★★★ | 9/9 | Included |
| Yanagi 2021^a^ | ★★★☆ | ★☆ | ★★★ | 7/9 | Included |
| Yao 2021^a^ | ★★★★ | ★★ | ★★★ | 9/9 | Included |

***Note.*** ^a^Cohort: Scores are 0–4 stars for selection, 0–2 stars for comparability, and 0–3 stars for outcome. Studies scored 0 or 1 star in selection domain, or 0 stars in comparability domain, or 0 or 1 stars in outcome/exposure domain were considered having poor methodological quality; ^b^Cross-sectional: Scores are 0–5 stars for selection, 0–2 stars for comparability, and 0–3 stars for outcome. Studies scored below 4 of the total score were considered having unsatisfactory methodological quality.


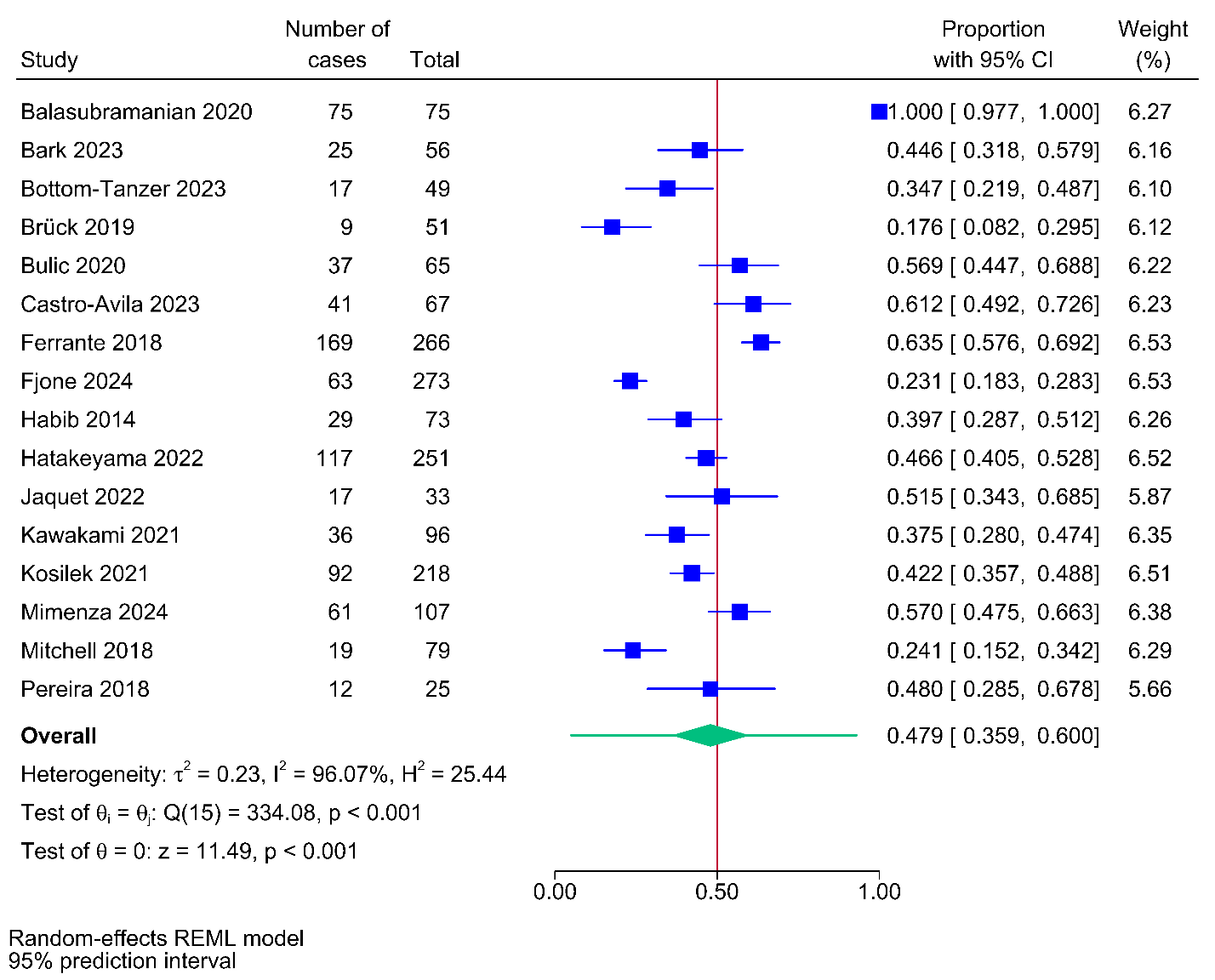


**eFigure 1.** Pooled Proportions of Post-Intensive Care Cognitive Impairment at 4 to 6 Months Follow-up


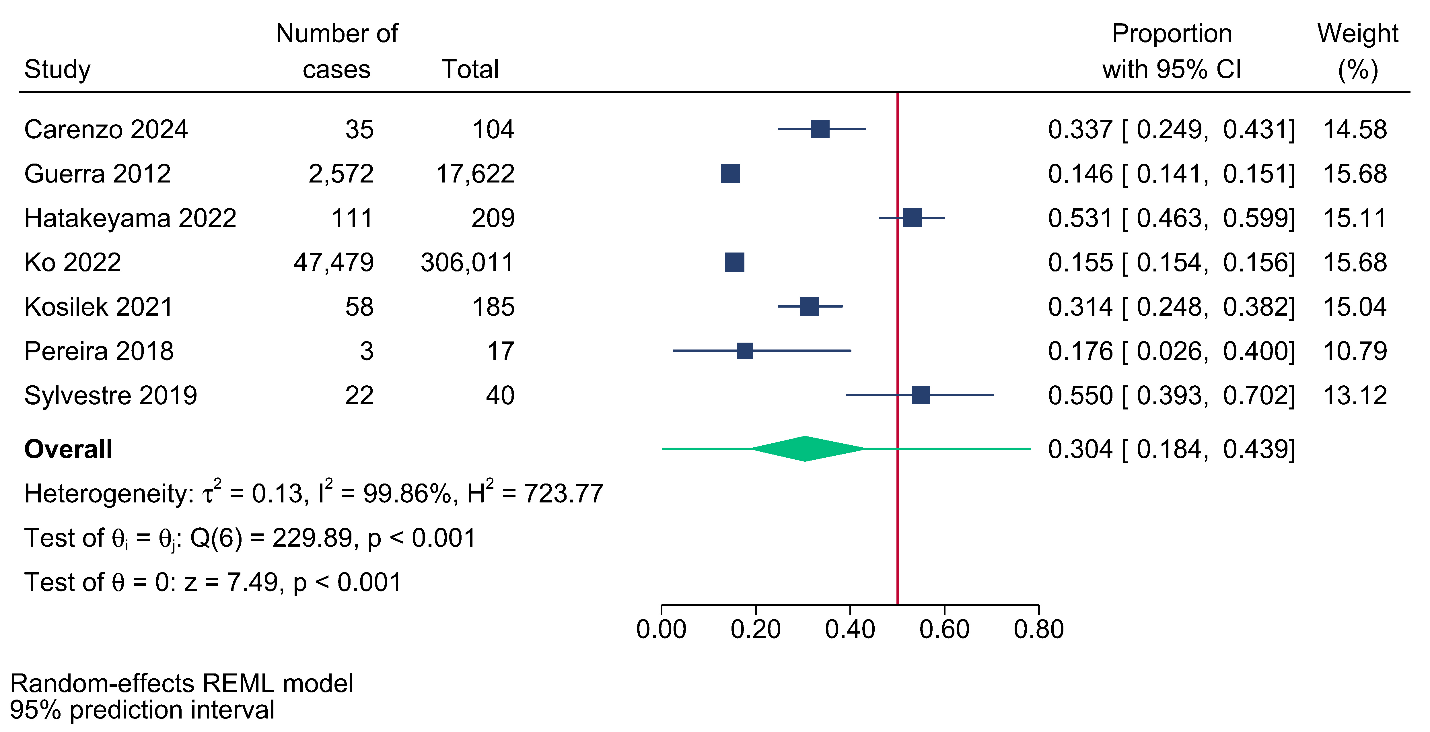


**eFigure 2.** Pooled Proportions of Post-Intensive Care Cognitive Impairment at over 12 Months Follow-up

**
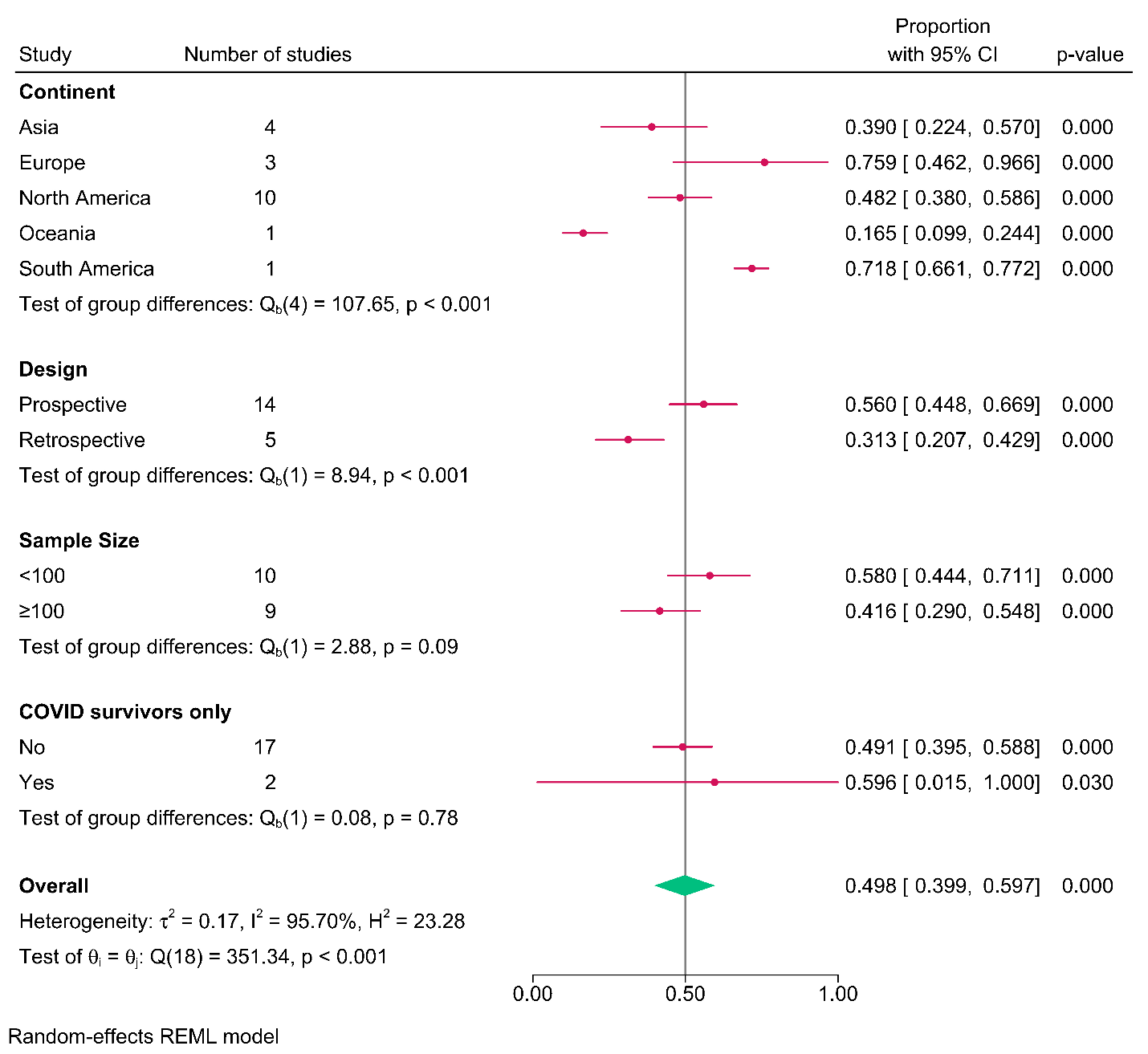
**

**eFigure 3.** Subgroup Analyses on Post-Intensive Care Cognitive Impairment Prevalence at Follow-Up Within a Month

**
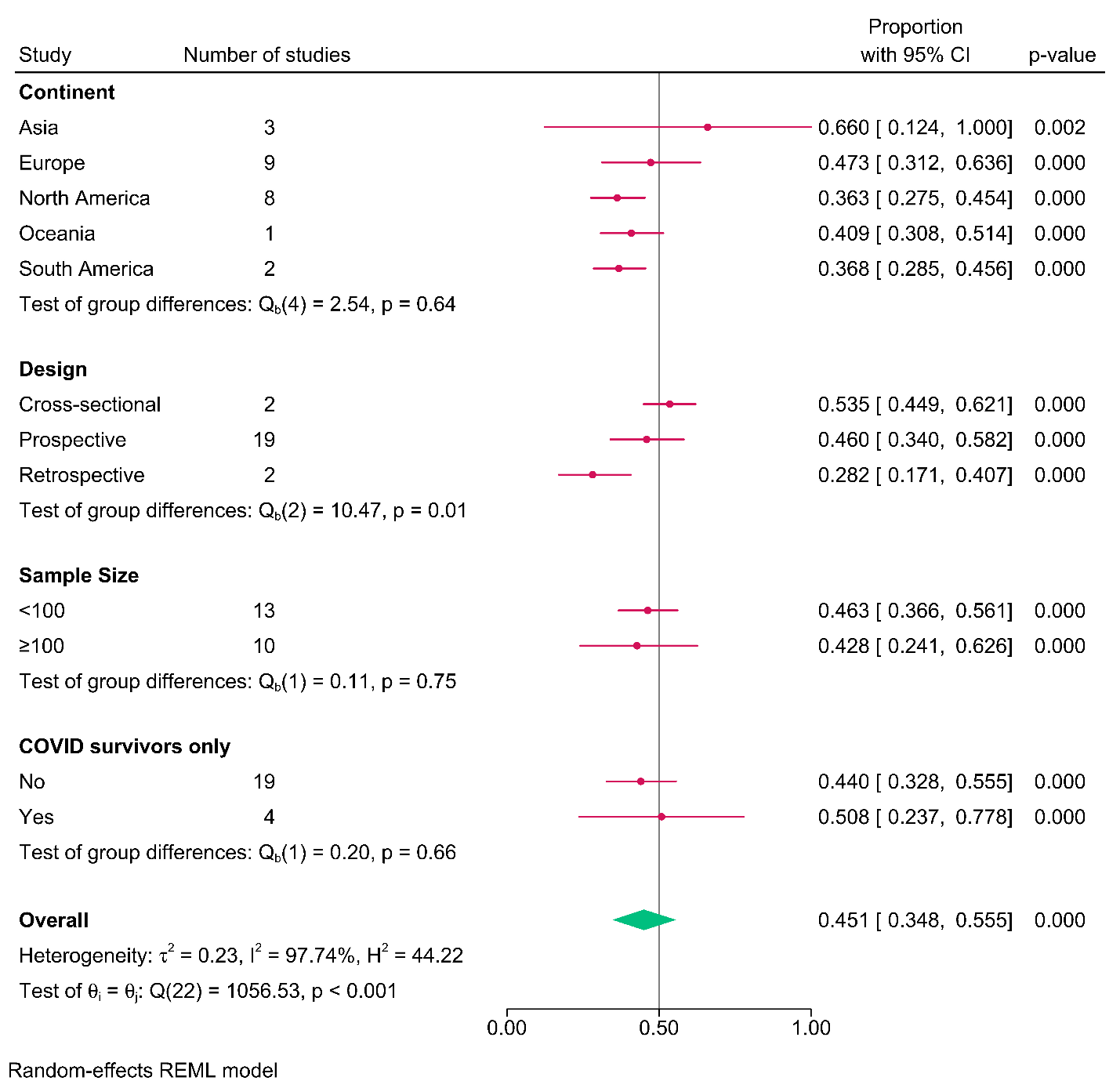
**

**eFigure 4.** Subgroup Analyses on Post-Intensive Care Cognitive Impairment Prevalence at 1 to 3 Month(s) Follow-Up

**
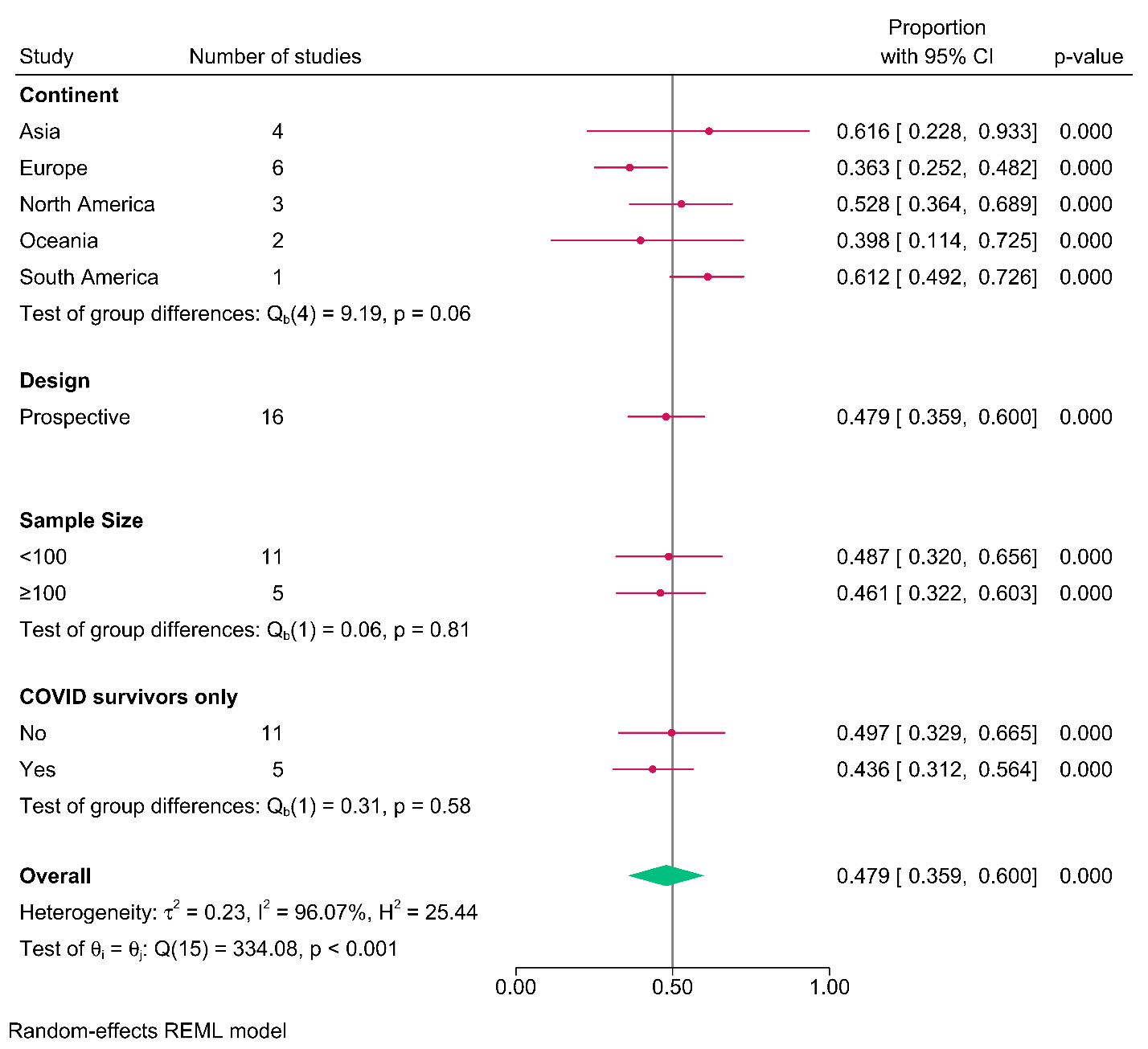
**

**eFigure 5.** Subgroup Analyses on Post-Intensive Care Cognitive Impairment Prevalence at 4 to 6 Months Follow-Up

**
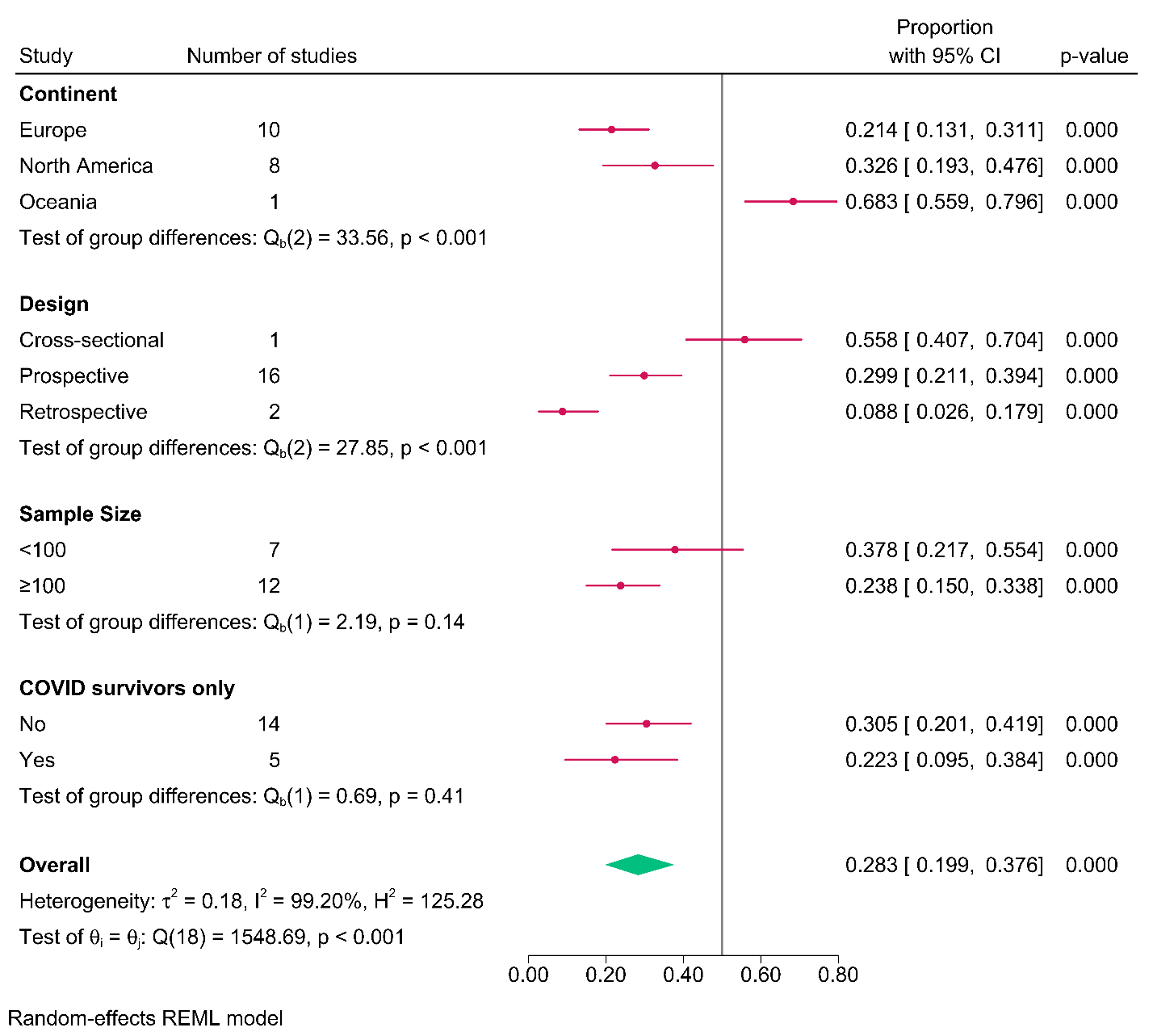
**

**eFigure 6.** Subgroup Analyses on Post-Intensive Care Cognitive Impairment Prevalence at 7 to 12 Months Follow-Up

**
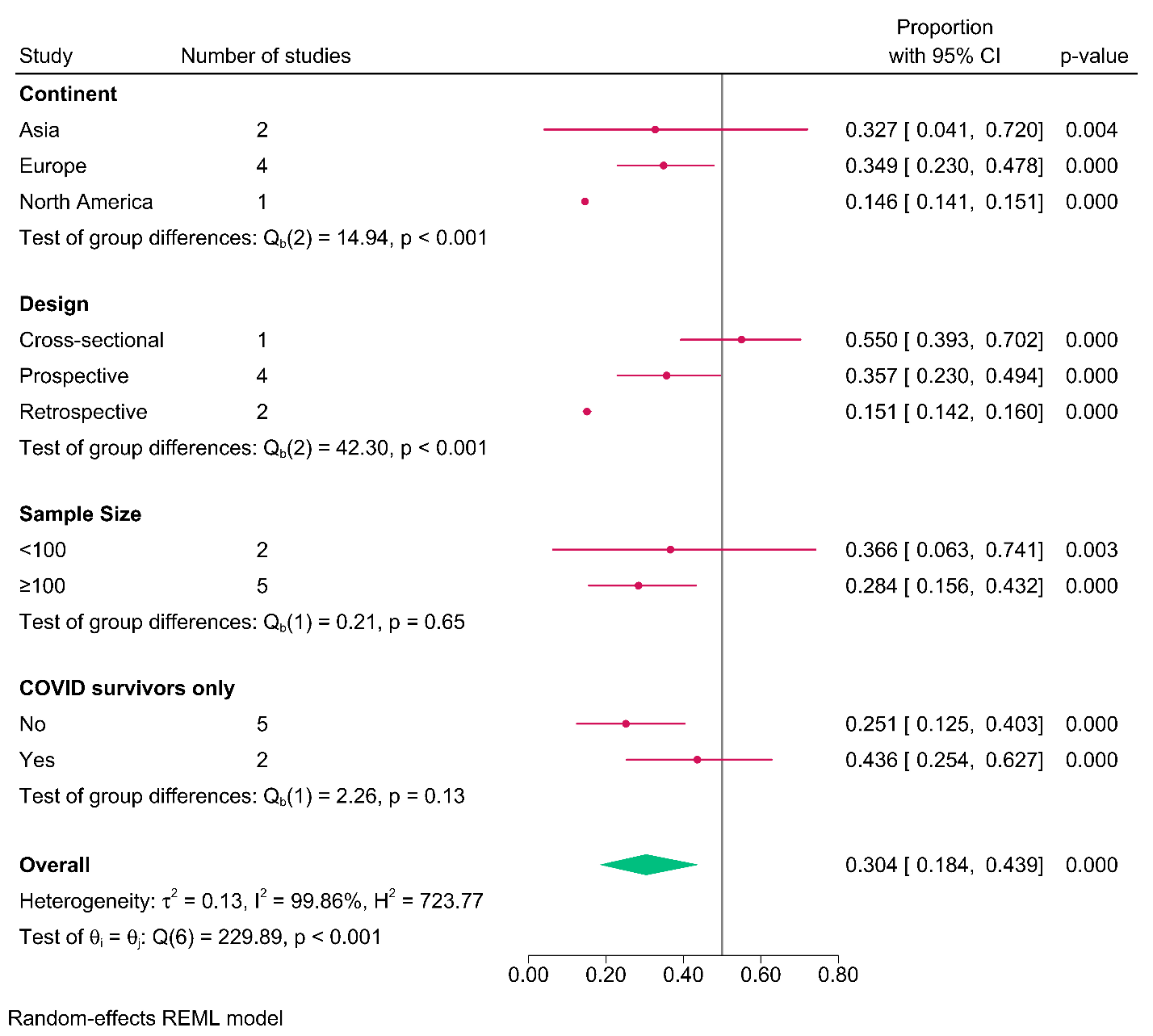
**

**eFigure 7.** Subgroup Analyses on Post-Intensive Care Cognitive Impairment Prevalence at Follow-Up Over 12 Month

**
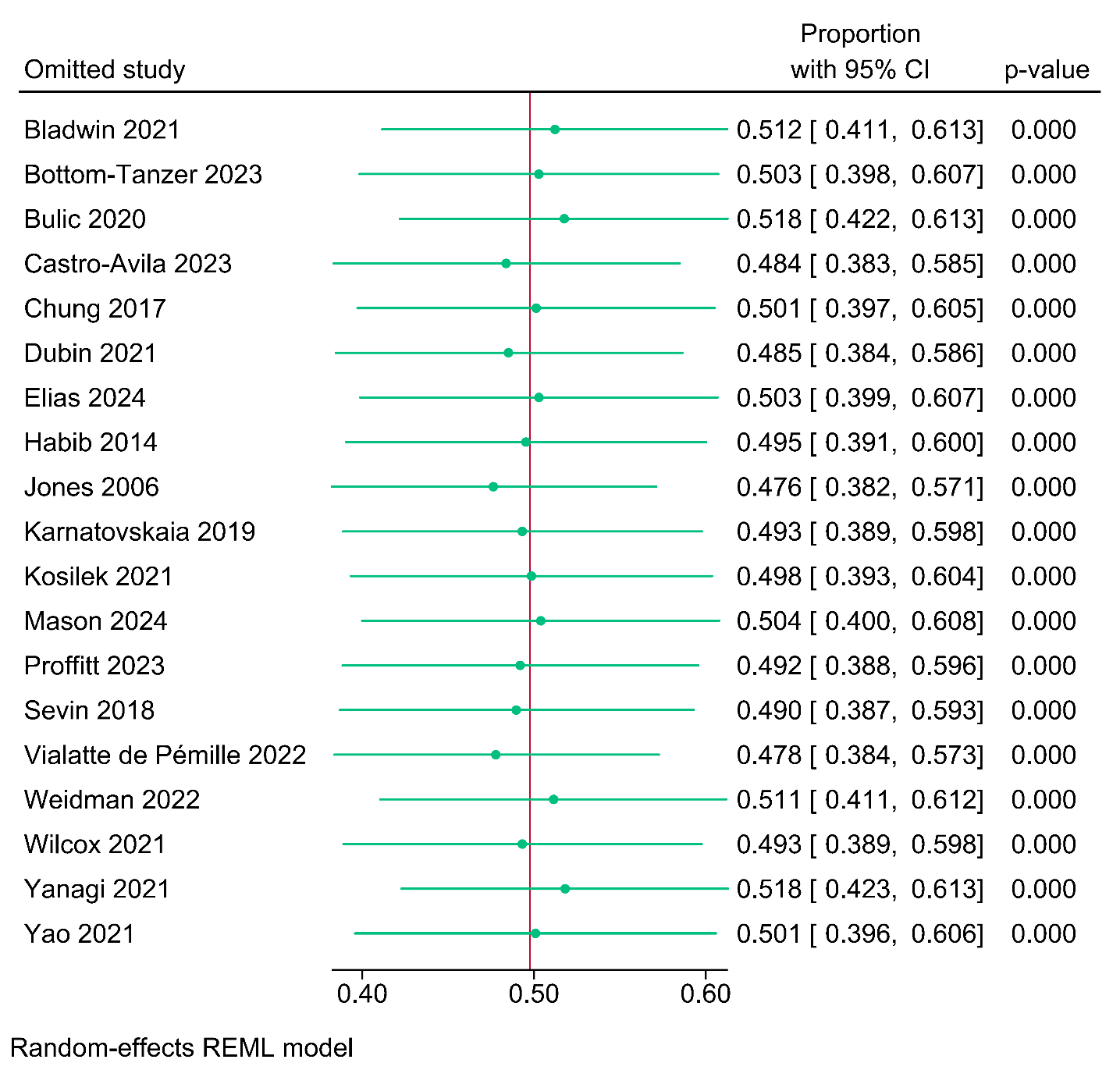
**

**eFigure 8.** Leave-One-Out Meta-Analysis on Post-Intensive Care Cognitive Impairment Prevalence at Follow-Up Within a Month

**
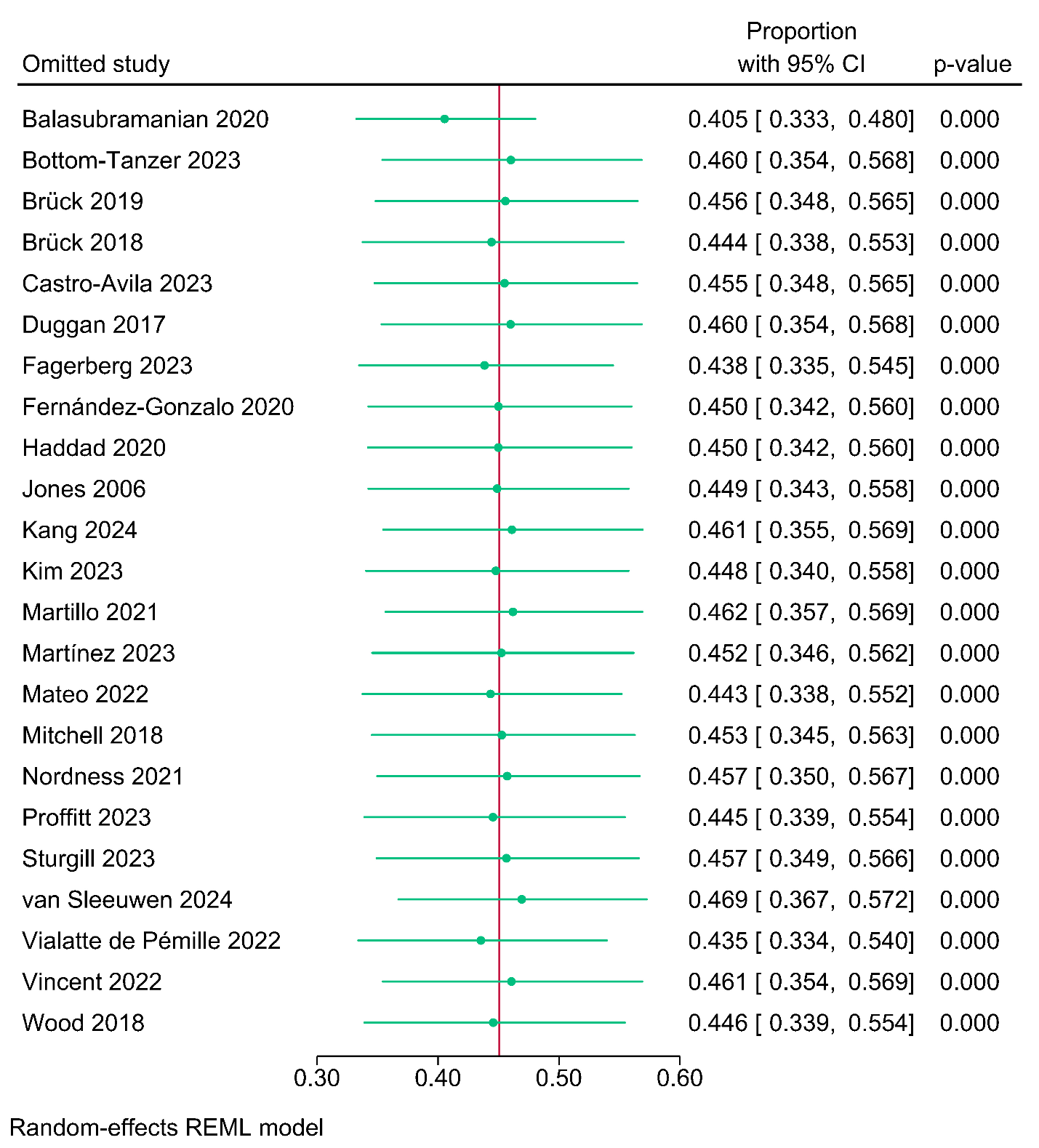
**

**eFigure 9.** Leave-One-Out Meta-Analysis on Post-Intensive Care Cognitive Impairment Prevalence at 1 to 3 Month(s) Follow-Up

**
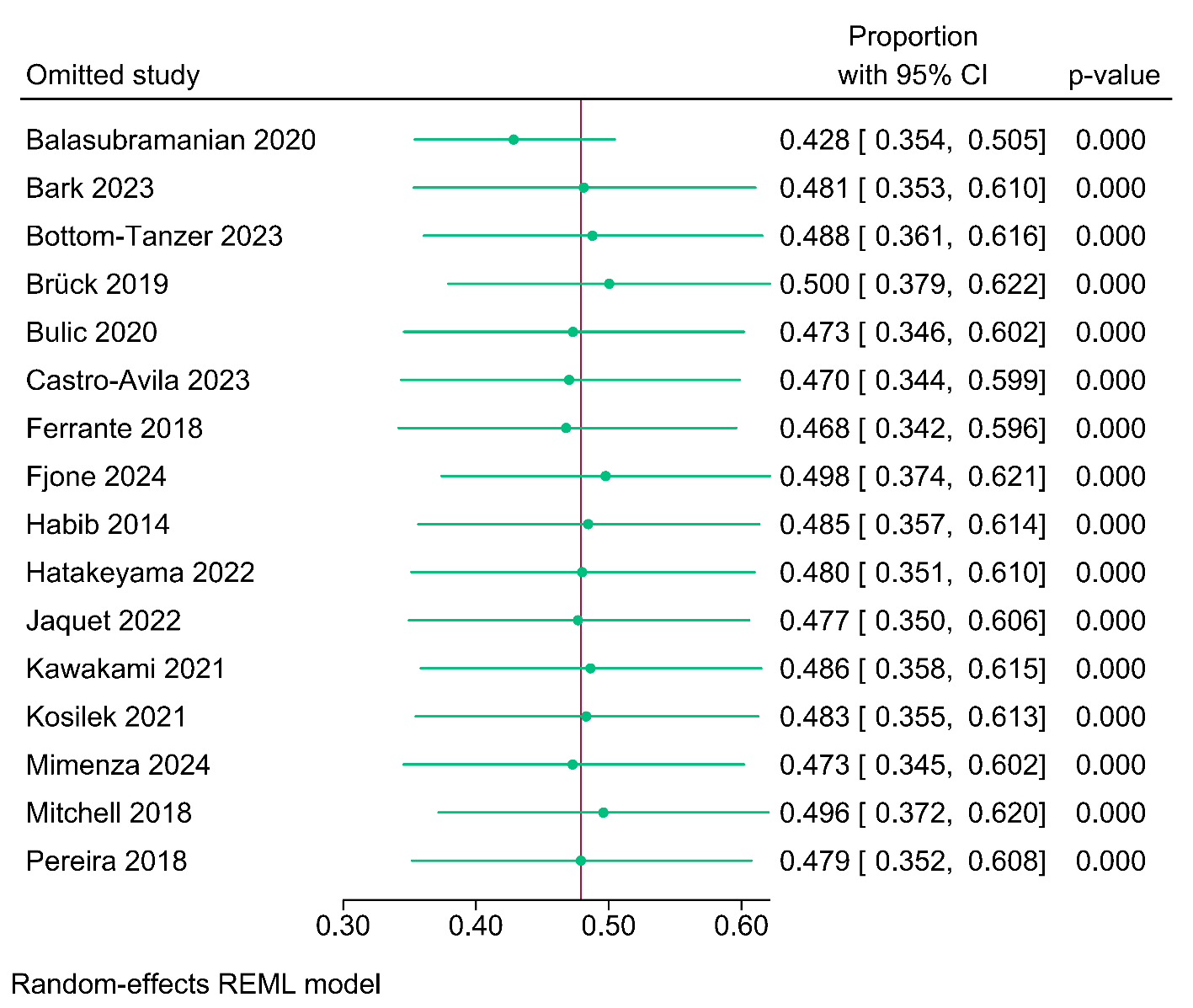
**

**eFigure 10.** Leave-One-Out Meta-Analysis on Post-Intensive Care Cognitive Impairment Prevalence at 4 to 6 Months Follow-Up

**
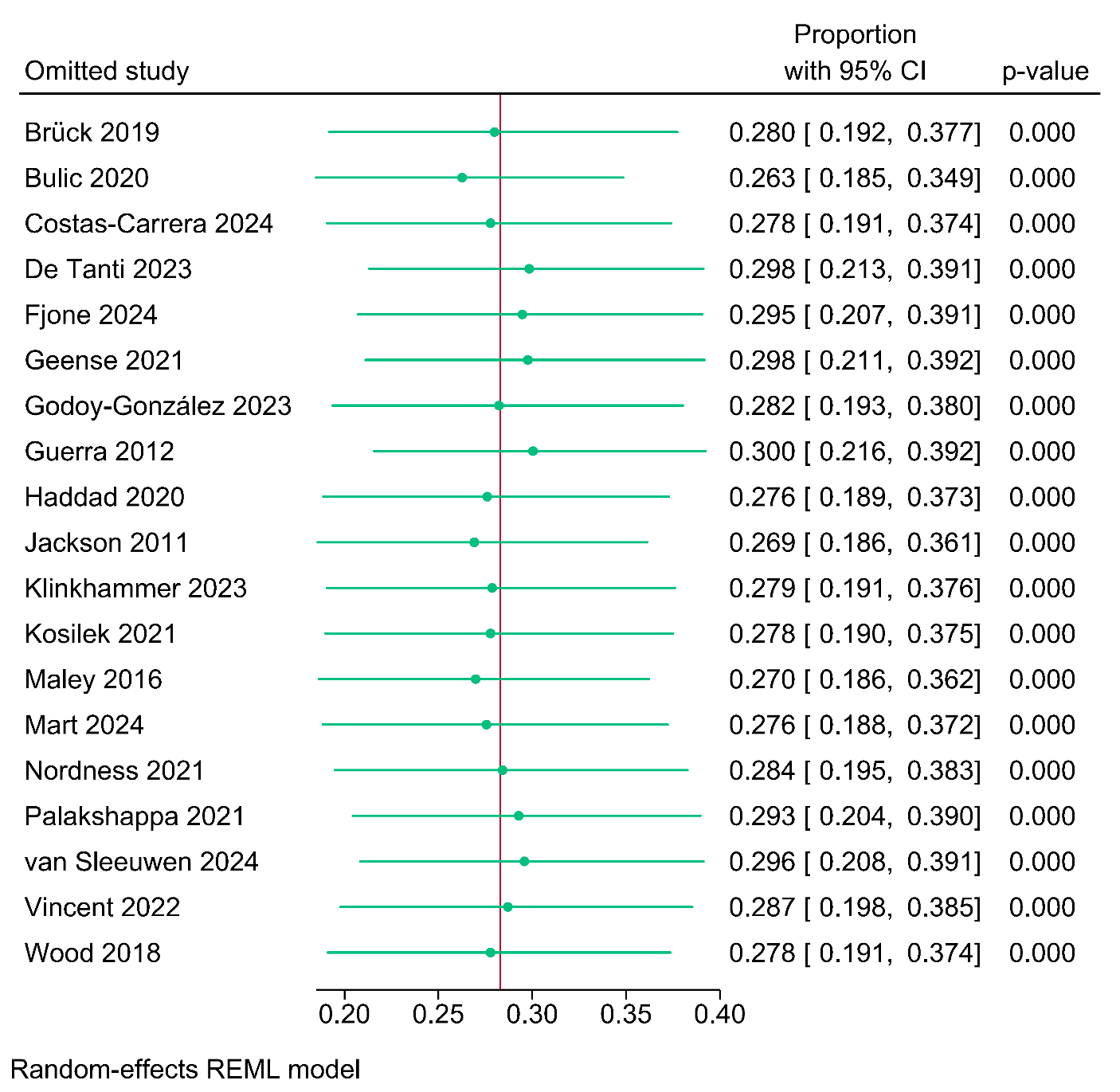
**

**eFigure 11.** Leave-One-Out Meta-Analysis on Post-Intensive Care Cognitive Impairment Prevalence at 7 to 12 Months Follow-Up

**
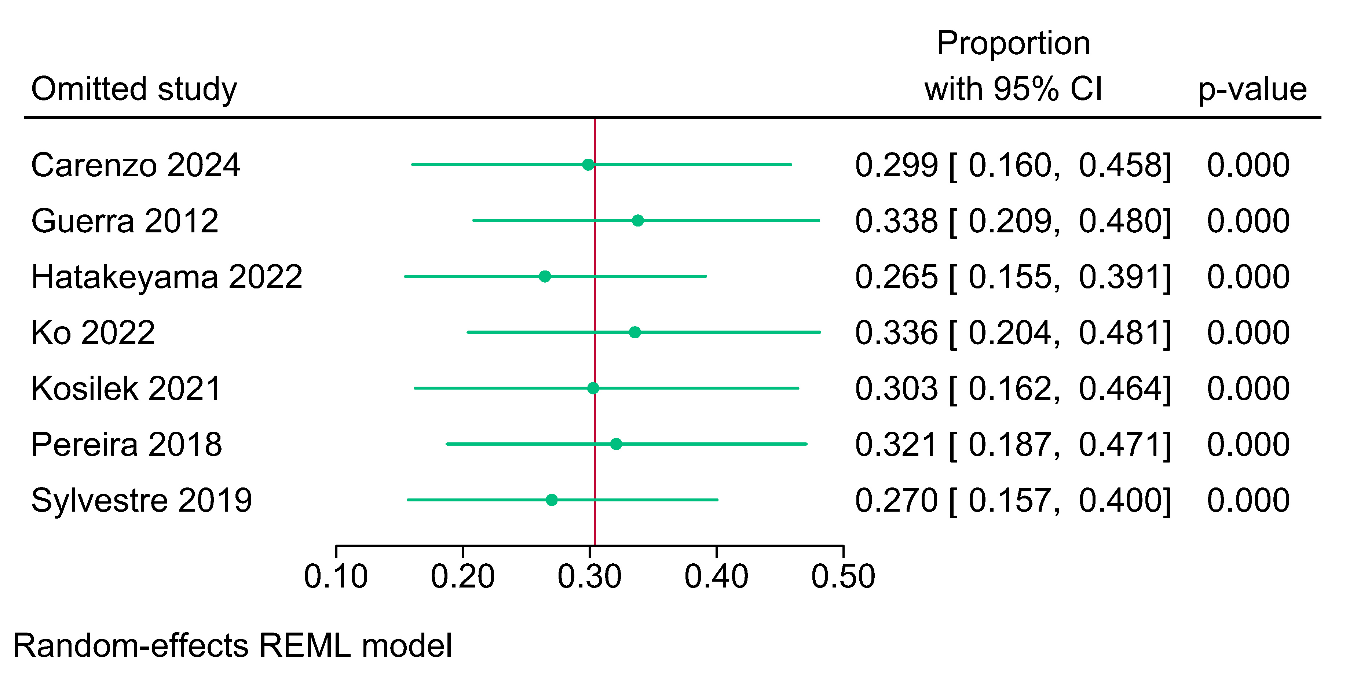
**

**eFigure 12.** Leave-One-Out Meta-Analysis on Post-Intensive Care Cognitive Impairment Prevalence at Follow-Up Over 12 Months
